# Effectiveness and acceptance of virtual reality vs. traditional exercise in obese adults: a pilot randomized trial

**DOI:** 10.3389/fspor.2025.1520068

**Published:** 2025-03-19

**Authors:** Evlalia Touloudi, Mary Hassandra, Evangelos Galanis, Gerasimos Pinnas, Charalampos Krommidas, Marios Goudas, Yannis Theodorakis

**Affiliations:** Department of Physical Education and Sport Sciences, University of Thessaly, Trikala, Greece

**Keywords:** obesity, VR, exercise, behavioral change, counseling, SDT

## Abstract

**Introduction:**

Obesity is a significant global health concern, increasing the risk of various diseases and health complications. Effective and engaging exercise interventions are urgently needed for obesity management. This pilot study examines the effectiveness of virtual reality (VR)-based exercise compared to traditional exercise, both combined with Self-Determination Theory (SDT)-based counseling, individuals with obesity.

**Methods:**

Forty individuals with overweight/obesity were randomly assigned to either a VR-based exercise group (intervention) or a traditional exercise group (control), with both groups receiving SDT-based counseling over 4 weeks. Assessments were conducted pre- and post-intervention.

**Results:**

Participants in the VR-based exercise group showed significant reductions in BMI (2.6%) and body fat mass (5.3%) and consequently in hips circumference, compared to the control group. They also exhibited greater increases in physical activity levels, and improved psychological outcomes, including basic psychological needs (BPN), self-efficacy, self-esteem, and attitudes toward the program comparing with the control group. Moreover, the intervention group scored higher in interest/enjoyment and attitudes towards exercise compared with the control group and showed great results in perceived enjoyment, intention for future use, usability, and positive perceptions of using the VR system. Based on the participants’ interviews, VR-based exercise was found to be more enjoyable and engaging.

**Discussion:**

These findings suggest that VR-based exercise combined with counseling is more effective than traditional exercise in reducing weight, increasing physical activity, and improving psychological outcomes individuals with obesity. The immersive nature of VR may enhance motivation and adherence to exercise programs, offering a promising alternative for obesity management. However, the study's small sample size, short duration, and reliance on self-reported measures may limit its generalizability. Future research should explore long-term adherence, effectiveness in diverse populations, psychological mechanisms behind engagement, and comparisons with emerging fitness technologies like AI-powered platforms.

## 1 Introduction

Obesity is a major risk factor for numerous noncommunicable diseases (NCDs), such as diabetes, cardiovascular diseases, and certain types of cancer (1). It is also associated with numerous mental health issues like depression, poor quality of life and body dissatisfaction. Many individuals with obesity experience psychological distress due to being subjected to weight stigma and discrimination. Given these serious health implications, effective management strategies for obesity are crucial (2–4). The role of exercise programs and physical activity is crucial, as they are key components in the treatment of obesity. Beyond promoting weight loss through increased energy expenditure, physical activity also enhances overall metabolic and cardiovascular health. It can lead to significant improvements in biomarkers for people with obesity, such as lower serum triglycerides and reduced arterial stiffness. Participation in exercise programs can also lead to improved cardiorespiratory health muscle strength, quality of life, and sustained exercise behavior. Additionally, physical activity offers notable benefits for brain health, including better sleep quality and reduced depressive symptoms. It also positively impacts emotional well-being and overall quality of life (5). Individuals can engage in various forms of exercise to manage obesity. Programs incorporating moderate-intensity aerobic exercise and moderate-to-high intensity resistance training are particularly recommended for adults aiming to achieve these health goals (6). Other researchers support that high-intensity interval training (HIIT) can significantly improve mental health-related indices in overweight and obese adults, with improvements equal to or greater than those observed for moderate-intensity continuous training (7). Nonetheless, regular exercise and healthy nutrition contribute to weight control, disease prevention, maintenance, and metabolic fitness in obesity, with endurance training being the most effective (8, 9).

Effective obesity management requires a multifactorial approach that addresses not only diet and exercise but also psychological and behavioral factors to foster long-term change (10). Multi-component interventions that incorporate behavioral modification, cognitive strategies, and values-based approaches have been shown to enhance adherence, confidence, and intrinsic motivation, leading to improved health outcomes (11, 12). Individualized nutrition counseling plays a crucial role, influencing both physical and mental well-being, as it has been linked to reduced depressive symptoms and sustained weight loss (13). Obesity is often linked to lower self-esteem, with studies highlighting a negative relationship between excess weight and body image (4, 14). A positive body image and a flexible approach to eating restraint have been associated with better weight control outcomes (15). Similarly, self-efficacy—one's confidence in their ability to adopt healthy behaviors—has been identified as a strong predictor of successful weight management and increased physical activity, enhancing adherence to dietary and exercise guidelines (16). Self-regulation skills, such as self-monitoring, also contribute to weight loss success, with research showing a direct link between self-monitoring and higher weight loss rates in individuals with obesity (17). Additionally, positive attitudes toward nutrition and physical activity are key determinants of long-term behavioral change, reinforcing intention, perceived control, and engagement in healthy habits (18, 19). Among behavioral strategies, goal setting has been recognized as an effective tool in obesity management, with studies demonstrating its role in increasing effort and commitment to physical activity (20). Recent findings highlight the importance of setting Specific, Measurable, Achievable, Relevant, and Time-bound (SMART) goals, which have been positively associated with weight loss outcomes in community-based programs (21). In clinical settings, goal-setting has been successfully applied in cardiac rehabilitation and nutrition interventions, further emphasizing its relevance to weight management (22, 23). Therefore, counseling, behavioral change strategies, and traditional exercise interventions are beneficial, and in recent years, innovative approaches like Virtual Reality (VR) have shown promise in improving obesity management outcomes.

VR is a computer-generated simulation of a real or imaginary three-dimensional environment that allows users to explore and interact with it. Over recent decades, VR has expanded beyond entertainment and leisure, finding applications in exercise, education, rehabilitation, health, and various other fields [(24) accessed on 8 July 2024]. Several studies have explored the effects of VR exercise and support the idea that it is suitable for yielding positive outcomes in physiological, psychological, and recovery-related factors when compared to traditional exercise (25). A systematic review found that VR technology has great potential for assessing and treating obesity due to its interactivity, flexibility, and customization to individual needs, making it compatible with existing psychological theories and practices (26). Research shows that people tend to respond to virtual environments as if they were real, and individuals generally accept this technology (27). A study found that VR self-conversations based on motivational interviewing can help individuals with obesity improve habit change readiness, self-efficacy, and reduce dysfunctional eating behaviors and anxiety (28). VR, when combined with cognitive behavioral therapy or weight loss programs, supports significant weight loss, though maintaining it is challenging (26). Another study suggests that VR integration into behavioral weight loss treatments may help sustain weight loss (29). Additionally, incorporating VR into physical activity programs could counteract reduced engagement due to weight-based victimization (30).

Despite the growing use of VR in health interventions, little research has examined its combination with Self-Determination Theory (SDT)-based counseling for obesity management. SDT posits that motivation and well-being are enhanced when individuals experience autonomy, competence, and social support, which are crucial for sustaining behavioral changes (31, 32). In obesity treatment, SDT-based interventions have shown positive effects on promotion of health behavioral change like physical activity adherence, and psychological well-being (33, 34). While existing studies have demonstrated encouraging results, most have focused on VR or counseling as standalone approaches. VR interventions have been effective in promoting physical activity, improving self-efficacy and self-esteem, managing psychological distress and supporting weight loss (26, 35, 36). Furthermore, research suggests that VR reduces perceived exertion, making exercise feel easier and potentially increasing engagement in physical activity (37). Additionally, VR interventions combined with cognitive-behavioral techniques have improved motivation and self-esteem while reducing disordered eating behaviors (38). However, the potential combination of VR-based exercise and SDT-based counseling remains largely unexplored. It is highlighted the importance to address this gap by the evaluation of the combined effects of VR exercise and SDT-based counseling on physical activity, psychological well-being, and behavioral outcomes in individuals with obesity. Given that obesity management requires sustainable behavioral modifications (e.g., eating habits, physical activity, medication adherence), integrating behavioral change strategies—such as SDT-based motivation—with VR's engaging and immersive exercise experience may provide a more holistic and effective approach (11).

For the management of obesity, a combination of exercise and counseling appears to be effective. Additionally, individuals can exercise using VR systems and respond to virtual stimuli in a way that closely mirrors real-life experiences (39). However, there are few studies in the literature that have examined the impact of VR-based exercise combined with SDT-based counseling in individuals with obesity. VR systems have been successfully used and well-accepted by obese individuals to change behaviors and lifestyles for combating obesity through virtual self-talk and psycho-educational counseling (28). It is supported that it effectively improves body image satisfaction and promotes health self-efficacy individuals with overweight or obesity through self-monitoring of diet, physical activity, and weight (26). Additionally, according to a study involving overweight women, it was found that exercise in a virtual environment had a positive effect on exercise enjoyment, immersion in exercise, reduction in BMI, and depressive symptoms (40) Lastly, according to a systematic review, exercise in a virtual reality environment has been shown to have a positive impact and is a promising tool for managing smoking, diet, obesity, and increasing physical activity levels (41).

The primary aim of this study was to evaluate the impact of a VR-based exercise intervention combined with SDT-based counseling on key anthropometric, psychological and behavioral outcomes in individuals with overweight and obesity. Specifically, the study compared changes in BMI, body fat mass, basic psychological needs, attitudes towards the program, self-esteem, self-efficacy, and physical activity engagement between participants performing VR-based exercise and those engaging in traditional, self-selected exercise paired with SDT-based counseling. The secondary aim was to assess participants’ attitudes, interest, and enjoyment regarding each exercise modality, as well as the acceptance, usability, and overall enjoyment of the VR exercise platform.

## 2 Methods and materials

### 2.1 Trial design

This study followed a Randomized Controlled Trial (RCT) design with a control group. The study protocol was approved by the Institution Ethics Committee (Approval No. 1829, 13 October 2021).

### 2.2 Participants

In our study, 40 individuals with overweight/obesity (24 female and 16 male) with a BMI > 25 kg/m^2^, aged from 18 to 65 years old participated. Demographic characteristics of the participants are presented in Table 1. All participants were informed about the study's objectives and signed the consent form for participation in research studies. Two groups of 20 individuals each were assigned: (a) VR exercise group, and (b) No VR exercise group (control group). The randomization process proceeded as follows: The first participant was randomly assigned to a group using a coin flip. Following this, participants were alternately assigned to the experimental and control groups. Specifically, after the initial random assignment, one participant was allocated to the experimental group, while the next was assigned to the control group, continuing this alternating pattern until the desired number of participants was reached.

**Table 1:** Demographic characteristics and anthropometric values.

Variable	Units	Group	Mean ± SD	Frequency
Age	years	Intervention	40.60 ± 11.93	–
		Control	45.65 ± 12.17	–
Height	m	Intervention	1.67 ± 0.10	–
		Control	1.73 ± 0.08	–
Weight	kg	Intervention	90.36 ± 16.69	–
		Control	97.86 ± 11.54	–
BMI	kg/m^2^	Intervention	32.69 ± 5.25	–
		Control	32.64 ± 2.70	–
Body fat mass	kg	Intervention	36.81 ± 9.47	–
		Control	37.40 ± 8.06	–
Gender	–	Intervention	–	30% male, 70% female
		Control	–	50% male, 50% female
Education	–	Intervention	–	Secondary 30%, Tertiary 70%
		Control	–	Secondary 25%, Tertiary 75%

#### 2.2.1 Recruitment—setting: community setting intervention

Participants were recruited through an open call to the community of Volos, an urban city in central Greece. This initiative aimed to inform local residents and provide them with the opportunity to express their interest in participating in the study. Then, more specifically, individuals were informed about the project and agreed to participate.

An informational brochure was created to inform the public about this initiative and encourage their participation (Supplementary Material 1). The brochure was published on social media and distributed in various crowded locations, including sports facilities. On the brochure, there was a QR code that directed them to an online participation form which included study inclusion and exclusion criteria such as age, weight, height, and if they have been diagnosed with neurological, orthopedic, cognitive, or psychiatric conditions, poor or no vision, and vertigo, as well as their contact details (phone number or email) to invite them to the first meeting.

### 2.3 Intervention

The intervention program was grounded in SDT to facilitate behavior change in participants with overweight and obesity. Based on the core principles of SDT, which include autonomy, relatedness, and competence, the following intervention was designed including exercising in VR environment combined with 3 counseling sessions to promote engagement in physical activity among individuals with overweight and obesity and guide them toward a healthier lifestyle for the intervention group. The behavior changes techniques (42) used and applied in the intervention for every need are presented in Supplementary Material 2.

### 2.4 Materials

#### 2.4.1 Brochure, forms and booklet

To cover the needs of the intervention, the following materials were created. An informational brochure about the existence of the VR exercise program and counseling, a consent form describing in detail the study procedures and ensuring personal data privacy according to GDPR (“I hereby consent to the collection, processing, and secure handling of my personal data in accordance with data privacy and protection regulations.”), a booklet that includes information and advice about exercise, diet and ways to overcome psychological difficulties in their efforts to systematically participate in exercise and nutrition programs (Supplementary Material 3) a Daily Self-Monitoring Form in which participants recorded daily step count, water intake, consumption of sugary foods, the quantity of fruits and vegetables eaten, and the duration of physical activity and a Goal-Setting Form in which participants recorded their goals for physical activity, nutrition, and water intake for the next week and complete the Implementation Intention Plan for each goal (Supplementary Material 4, 5) The goal-setting forms were completed after every counseling session and based on the goal-setting theory (43).

#### 2.4.2 VR exercise application

The VR exercise app used for the intervention program is called PowerBeats VR. PowerBeatsVR is an immersive VR exercise application designed to enhance physical activity through an engaging and interactive environment. Participants engage in a variety of workout routines that combine cardio and strength training exercises with rhythmic movements and music. All of them are transported into visually stimulating VR worlds, ranging from futuristic settings to natural landscapes (such as desert, sky, space, and medieval), which make the workout experience more enjoyable. The application offers a range of pre-designed workout routines catering to different fitness levels and goals. Participants can choose routines based on their preferences, focusing on cardio, strength, or a combination of both. The exercises are synchronized with energetic music, motivating participants to move in time with the beat. This feature not only makes the workout fun but also helps maintain a consistent exercise rhythm. During the workout, participants engage in interactive gameplay elements such as punching orbs, dodging obstacles, and performing various body movements. This gamified approach keeps participants engaged and focused throughout the session. The exercise sessions typically lasted for 15–60 minutes, with participants encouraged to engage in at least three sessions per week. The intensity of the workouts varied from moderate to vigorous, based on the participants’ fitness levels and preferences. Real-time feedback provided metrics such as calories burned and exercise duration, helping participants track their progress and stay motivated. Participants could set personal fitness goals within the app and monitor their achievements over time and have a personalized exercise experience throughout the intervention. The app kept a detailed record of completed workouts, allowing users to see their progress and make adjustments to their fitness plans as needed. PowerBeatsVR allowed participants to customize their workout sessions by selecting different difficulty levels, workout durations, and types of exercises, ensuring that the workout remained challenging and suitable for the participant's fitness level (Supplementary Material 6, 7).

#### 2.4.3 Counseling sessions

The 3 counseling sessions were designed to promote behavioral changes towards exercise, diet, and a healthy lifestyle. These sessions were provided to both groups at the beginning of the first week, the end of the second week, and the end of the fourth week of the intervention. Each session was based on the core principles of SDT, focusing on enhancing autonomy, relatedness, and competence. The content of the sessions included:

• Session 1: Introduction to the importance of physical activity and healthy eating, initial goal setting using SMART principles (Specific, Measurable, Achievable, Relevant, Time-bound)—a framework known to enhance goal effectiveness (44–46), and strategies to overcome psychological barriers.
• Session 2: Review of progress, adjustment of goals, strategies for maintaining motivation, and discussion on the importance of social support and relatedness.
• Session 3: Final evaluation of progress, setting long-term goals, strategies for maintaining behavior changes post-intervention, and discussion on competence and self-efficacy.

Goal-setting techniques were used throughout the sessions to help participants set and achieve both short-term and long-term goals related to daily steps, exercise duration, types of physical activities, nutrition choices, and water intake. The control group followed the counseling sessions without the opportunity to use the VR exercise system.

### 2.5 Procedures

After their categorization into the Intervention group (VR exercise) and Control group (No VR exercise), based on systematic allocation, all participants were contacted by phone, the study's procedure was explained and the first meeting was organized. All phases of the intervention (VR-based exercise sessions and counseling) were delivered by an exercise specialist with expertise in exercise psychology.

During the initial meeting, participants from both groups were informed in detail about the study phases, provided their consent, and completed the first questionnaire. Participants were informed about the aim of the study, that it was to evaluate the effects of each exercise intervention, in combination with counseling, on physical, psychological, and behavioral factors. However, individuals were not explicitly told that the primary focus was on comparing VR-based exercise with traditional exercise. This approach aimed to minimize potential bias in their responses and engagement levels. Each participant received printed Daily Self-Monitoring Forms to fill out daily for the following week. This session also included counseling, goal-setting, and the distribution of the booklet with information and advice (47). Participants completed the goal-setting form with their goals for the following week and were encouraged to complete the goal-setting form every week, check their achievements, and adjust their goals according to SMART goal-setting (44–46). Additionally, all, scheduled an appointment with our partner nutritionist for body measurements, including height, weight, waist, hip and neck circumference, and body fat mass.

Subsequently, only participants from the intervention group conducted a trial exercise session with the VR application, familiarizing themselves with the VR equipment, receiving detailed usage instructions, and having the opportunity to ask questions before starting the first VR exercise session. Safety measures were implemented to ensure participant well-being during the VR exercise sessions. To minimize the risk of motion sickness, participants were gradually introduced to the VR environment, starting with low-intensity movements and progressively increasing intensity based on individual tolerance. Additionally, regular breaks were encouraged, and participants could stop the session at any time if they experienced discomfort. The exercise specialist was present during all VR sessions to monitor participants, provide immediate assistance if needed, and ensure proper use of the equipment to prevent injuries.

From this point, both groups had 4 weeks to exercise, with the Intervention group having the option to use the VR system as an additional exercise method whenever desired. All participants from the Intervention group were allowed to exercise in the VR environment as much as they wanted, from 3 to 7 times per week. Participants arranged an appointment with the exercise specialist and had access to the VR device in an agreed convenient place (home or gym). The VR exercise was tailored to the individual fitness levels of each participant. Needed adjustments were made for participants who experienced physical discomfort or other constraints. These modifications ensured the safety and comfort of participants without compromising the overall intervention goals. Participants in the control group were free to engage in various forms of exercise, with an emphasis on aerobic activities such as cycling, swimming, gym-based group fitness classes (moderate to high intensity), and running. However, no structured exercise program was assigned, and no dietary intervention was implemented. The same counseling intervention as the experimental group was provided, but without the VR-based exercise component. Similarly, participants in the intervention group also had the option to engage in these types of exercises. Both the intervention and control groups had the flexibility to select preferred types of physical activity, ensuring that intensity ranged from moderate to vigorous. All participants were encouraged to engage in exercise at least three times per week, with each session lasting more than 30 minutes. Self-monitoring and progress tracking were also encouraged. Daily Self-Monitoring Forms were completed during the first, third, and final week of the program. Communication with the exercise specialist was maintained via Viber, providing the opportunity to discuss any issues—including barriers to physical activity faced by individuals with overweight and obesity—and seek solutions, instructions, or support regarding the exercise regimen. Additionally, general dietary tips for maintaining a healthy and balanced diet were available, but no structured dietary intervention was provided. Viber was used as a direct communication channel, allowing continuous support throughout the study. Whenever guidance, motivation, or clarification was needed, the exercise specialist was available. This real-time accessibility helped address concerns immediately, reinforce adherence to the intervention, and enhance accountability. The ability to maintain constant communication also fostered a sense of connection and encouragement, which is crucial for sustaining behavioral changes and promoting long-term engagement in physical activity. Participants had the opportunity to create small groups and exercise together. Both groups were free to choose their preferred way of exercising, selecting the hours and type of exercise according to individual schedules, goals, and advice from the exercise specialist. However, participation in an organized and structured exercise program was not required.

At the end of the second week, an additional meeting was held for counseling, discussion, and feedback on their progress. The participants completed once again the goal-setting form and set new goals for the following week. After the 4-week intervention, we conducted a final meeting where participants from both groups underwent body measurements again with our partner nutritionist, completed final questionnaires and interview, and attended the last counseling session. This session included discussions about their progress with the intervention and goal-setting for the future.

#### 2.5.1 Follow-Up

Four weeks post-intervention, participants from both groups were invited to complete a follow-up questionnaire assessing their adherence to exercise. Measures included self-reported physical activity levels and goal-setting progress. Additionally, participants were asked about their long-term intentions for physical activity, motivation levels, and any challenges they faced in maintaining their exercise routines post-intervention.

### 2.6 Measures

#### 2.6.1 Anthropometric measures

Each participant scheduled an individual appointment with our partner nutritionist at a convenient date and time. To ensure consistency, the follow-up assessment was conducted at approximately the same time of day as the initial measurement. Anthropometric assessments, including weight, body fat percentage, and fat mass, were conducted using the CHARDER MA601 body composition analyzer. Participants were instructed to follow standard pre-assessment guidelines, including avoiding food, caffeine, and vigorous physical activity for at least 3 hours prior to the measurement and emptying their bladder before the assessment to minimize variations due to fluid retention. Wearing light clothing and no shoes were encouraged to ensure accuracy. Height was measured using the Charder HM250U ultrasonic stadiometer.

#### 2.6.2 First meeting questionnaire

Both groups filled in questionnaires including questions about demographic characteristics (birth date, gender, occupation, educational level), use of technology [Personal Innovativeness questionnaire, (48)], psychological distress [GHQ-12, (49),], body image [BI-AAQ, (50)], self-efficacy [exercise self-efficac scale (51)], level of physical activity (Leisure-Time Exercise Questionnaire (LTEQ), scored using the formula: (9 × Vigorous) + (5 × Moderate) + (3 × Light) calculated and utilized as a Leisure Index Score, (52)], self-esteem [Rosenberg self-esteem scale, (53)], the degree to which participants’ psychological needs are met within an exercise context [BPNES, (54)], attitudes toward participation in the program, intention, perceived behavioral control and subjective norms [TPB Questionnaire, (55)].

#### 2.6.3 Last meeting questionnaire

Participants filled in questionnaires with questions about the level of physical activity (LTEQ), the degree to which participants’ psychological needs are met within an exercise context (BPNES), attitudes toward participation in the program, intention, perceived behavioral control and subjective norms (TPB Questionnaire), attitudes towards exercise(VR exercise for the Intervention group) (55), interest/enjoyment of exercise (VR exercise for the Intervention group) [IMI, (56)], self-efficacy, self-esteem, and a semi-structured interview with questions about their exercise (with the VR system for the Intervention group) and counseling experience, their adherence to the exercise program and if they achieved their goals, such as “How do you think your participation in the program helped you change your behavior regarding exercise and nutrition?” and “How do you intend to continue exercising in the future?”. Participants of the Intervention group also responded to questions about perceived enjoyment from the VR exercise program (57), intention for future use (55, 57), system usability [SUS, (58)] and VR equipment (59).

### 2.7 Retention strategies

In the study, participant retention strategies were employed to maintain the number of participants throughout the study. Some sample retention strategies that were used include:

- Communication: Regular communication with participants through Viber and encouraging them not to drop out of the process. The Viber chat was always active to discuss tips for adherence to the exercise program or diet, problems that may appear, and other issues including barriers of people with overweight or obesity towards physical activity and exercise.
- Self—Rewards: During the goal-setting process, each participant was encouraged to plan their goal and a reward for themselves upon successfully achieving their goal. For instance, if they manage to reach a daily step count of 8,000 for two weeks, they could treat themselves to a spa day as a reward.
- Encouraging communication and monitoring: Active engagement by monitoring participants’ involvement and addressing issues that arise promptly. Self-monitoring their daily exercise and nutrition is a nice way to enhance their behavior change effort.

The adherence to the intervention was monitored through the Daily Self-Monitoring Forms which were checked at the end of each week, and participants received regular feedback from the exercise specialist. Additionally, participants from the VR group were asked to track their VR exercise sessions through the application, which recorded metrics such as calories burned and session duration. The exercise specialist maintained regular communication with participants via Viber to address any barriers to participation and provide motivation.

### 2.8 Data analysis

For the quantitative data analysis, a power of 85% and confidence interval of 95% were adopted, with an estimated value for a type I error of 5% for the sample size calculation in this study. Descriptive statistics and frequencies were used for demographic characteristics and baseline measurements. The Kolmogorov–Smirnov Test was performed, confirming that the sample followed a normal distribution, and Mauchly's Test of Sphericity was conducted, indicating that the assumption of sphericity was also met. The two-way repeated measures ANOVA analysis was used to determine the influence of the intervention and differences between the two groups in all variables. The two-way repeated measures ANOVA analysis was used due to the controlled structure of the study, where all participants completed assessments at fixed time points, and there were no missing data. Correlation analysis was also conducted to examine the relationship between the baseline measures and pre-post-intervention measures. All statistical analyses were performed with IBM SPSS (Statistics Version 29) and the level of significance was set at *p* < .05.

Semi-structured interviews were audio-recorded and transcribed verbatim. The qualitative data were then analyzed using thematic analysis. Two independent researchers familiarized themselves with the transcripts by reading them multiple times and engaged in an inductive coding process to identify initial codes reflecting emerging patterns. These codes were collated into potential themes and iteratively refined through discussions to resolve any discrepancies, ensuring consistency and rigor. This systematic approach allowed us to identify key themes related to participants’ experiences, attitudes, and perceptions of both VR-based and traditional exercise interventions.

## 3 Results

### 3.1 Baseline measurements: personal innovativeness, general mental distress, body image

Descriptive statistics were applied to calculate the means and standard deviations for baseline measures of personal innovativeness, general mental health, and body image. The results are presented in Table 2. A correlation analysis was conducted and the results revealed several significant relations. Personal innovativeness was found to have a strong positive correlation with self-efficacy pre-intervention (r = .59, *p* < .001), a significant positive correlation with interest/enjoyment (IMI) (r = .42, *p* = .008) and with physical activity levels at follow-up (LTEQ) (r = .40, *p* = .010). General mental distress showed a significant positive correlation with body image (r = .31, *p* = .049). Finally, body image demonstrated a small positive correlation with physical activity levels at follow-up (LTEQ) (r = .33, *p* = .035).

**Table 2:** Psychometric scores at pre-intervention for each group.

Variable	Intervention group			Control group		
	Mean ± SD	Minimum	Maximum	Mean ± SD	Minimum	Maximum
Personal Innovativeness	4.11 ± .72	3.00	5.00	3.49 ± 1.02	1.25	5.00
General mental distress	4.3 ± 2.87	1.00	10.00	4.95 ± 3.47	0	11.00
Body Image	4.13 ± 1.61	1.30	7.00	2.86 ± 1.34	1.0	5.10

### 3.2 BMI & body fat mass

A two-way repeated measures ANOVA was conducted to examine the differences in BMI and body fat between the intervention and control groups for two measurements (pre- and post-intervention). The results revealed a statistically significant group-time interaction for both BMI F(1,38) = 30.89, *p* < .001, 95% CI [30.39,34.13], and body fat F(1,38) = 28.35, *p* < .001, 95% CI [31.86,39.80]. Additionally, there were statistically significant differences between the pre-intervention and post-intervention measurements within the intervention group for both BMI F(1,38) = 79.15, *p* < .001 95% CI [pre: 30.796, 34.574; post: 29.983, 33.687] and body fat F(1,38) = 68.40, *p* < .001 95% CI [pre: 32.83, 40.79; post: 30.88, 38.83], with post-intervention scores being lower than pre-intervention scores. However, no statistically significant differences in BMI were found between the two groups at either pre- F(1,38) = .001, *p* = .97, or post-intervention measurement F(1,38) = .30, *p* = .59. Similarly, no statistically significant differences in body fat were observed between the two groups at either pre- F(1,38) = .04, *p* = .84 or post-intervention measurement F(1,38) = .73, *p* = .40. The means and SD are presented in Table 3.

**Table 3:** Statistics at post intervention for each group.

Variable	Intervention group			Control group		
	Mean ± SD pre	Mean ± SD post	Cohen's d	Mean ± SD pre	Mean ± SD post	Cohen's d
BMI (kg/m^2^)	32.69 ± 5.25	31.84 ± 5.08**	0.16	32.64 ± 2.71	32.54 ± 2.77	0.04
Body fat mass (kg)	36.81 ± 9.47	34.86 ± 9.28**	0.21	37.40 ± 8.06	37.22 ± 8.25	0.02
Body fat (%)	39.75 ± 6.03	39.09 ± 6.58	0.10	38.05 ± 6.12	37.97 ± 6.10	0.01
Hips (cm)	114.45 ± 6.00	112.80 ± 5.88**	0.28	113.85 ± 7.29	113.68 ± 7.19	0.02
BPN	4.09 ± 0.77	4.53 ± 0.52*	0.67	3.95 ± 0.54	4.08 ± 0.58	0.23
Autonomy	4.14 ± 0.83	4.48 ± 0.68	0.45	3.95 ± 0.55	4.11 ± 0.69	0.26
Competence	4.00 ± 0.81	4.44 ± 0.48*	0.59	3.85 ± 0.75	3.94 ± 0.67	0.13
Relatedness	4.11 ± 0.77	4.64 ± 0.63*	0.75	3.99 ± 0.81	4.20 ± 0.74	0.27
Attitudes toward the program	6.40 ± 0.68	6.92 ± 0.23*	1.02	5.96 ± 1.02	6.34 ± 0.80	0.41
Intentions to exercise	6.21 ± 1.01	6.55 ± 0.73	0.39	5.77 ± 1.50	6.05 ± 1.17	0.21
Perceived control	5.66 ± 1.28	6.14 ± 1.02	0.41	5.09 ± 1.59	5.35 ± 1.67	0.16
Subjective norms	6.68 ± 0.57	6.75 ± 0.53	0.13	6.30 ± 1.03	6.5 ± 0.76	0.22
Self-esteem	42.60 ± 5.20	45.20 ± 3.74*	0.57	42.80 ± 4.96	43.80 ± 3.35	0.24
Self-efficacy	24.95 ± 7.56	29.70 ± 5.39*	0.72	23.85 ± 7.03	25.75 ± 7.25	0.27
Physical activity	43.10 ± 31.42	65.15 ± 22.24*	0.81	37.80 ± 24.35	43.00 ± 24.59	0.21

Physical activity classification: active (≥24), moderately active (14–23), insufficiently active (<14).

* *p* < 0.05.

** *p* < 0.01.

### 3.3 Hips circumference

A two-way repeated measures ANOVA was performed to examine the differences in the variable hips circumference between the intervention and control group for two measurements (pre- and post-intervention). The results revealed that there was a statistically significant group-time interaction F(1,38) = 45.46, *p* < .001. There were also statistically significant differences between the pre-intervention and post-intervention measurements of the hip circumference in the intervention group F(1,38) = 113.77, *p* < .001 with the post-intervention scores being lower than the pre-intervention. However, there were no statistically significant differences between the two groups neither pre- (1,38) = .081, *p* = .778 nor post-intervention F(1,38) = .178, *p* = .676. There were no statistically significant differences in waist and neck circumferences observed. The means and SD are presented in Table 3.

### 3.4 Basic psychological needs, attitudes toward the program and intentions to exercise, self-efficacy & self-esteem

A two-way repeated measures ANOVA examined the differences in Basic Psychological Needs (BPN) (Autonomy, Competence, Relatedness), Attitudes toward the program and intentions to exercise, self-efficacy, and self-esteem between the intervention and control groups across pre- and post-intervention measurements. For BPN, no significant group-time interaction was found, but the intervention group scored significantly higher post-intervention compared to the control group [F(1, 38) = 6.74, *p* = .050] 95% CI [3.81,4.23], with significant pre-post improvement in the intervention group [F(1, 38) = 8.19, *p* = .013] 95% CI [4.28,4.78]. In Autonomy, Competence, and Relatedness, no significant group-time interactions were observed. Competence showed significant group differences at the second measurement [F(1, 38) = 7.33, *p* = .010], 95% CI [3.67,4.70] and significant pre-post improvement in the intervention group [F(1, 38) = 4.46, *p* = .041], 95% CI [pre: 3.65,4.35; post: 4.17,4.70]. Relatedness also improved significantly pre-post in the intervention group [F(1, 38) = 8.41, *p* = .006], 95% CI [pre: 3.76,4.47; post: 4.33,4.95]. Attitudes toward the program, as per the Theory of Planned Behavior, showed no group-time interaction but significant group differences post-intervention [F(1, 38) = 9.81, *p* = .003], 95% CI [6.44,6.81] and significant pre-post improvement in the intervention group [F(1, 38) = 4.68, *p* = .037] 95% CI [pre: 6.01,6.79; post: 6.65,7.18]. No significant differences were found in other TPB components. For self-efficacy and self-esteem, no significant group-time interactions were noted, but both showed significant pre-post improvement within the intervention group (F(1, 38) = 5.50, *p* = .024, 95% CI [pre: 21.68,28.25, post: 26.81,32.59] and F(1, 38) = 4.35, *p* = .044, 95% CI [pre: 40.30,44.90, post: 43.59,46.81], respectively). The means and SD are presented in Table 3.

### 3.5 Physical activity level

A two-way repeated measures ANOVA was performed to examine the differences in the variable physical activity between the intervention and control group for three measurements (pre-, post-, and one month after the intervention). The results revealed that there was no statistically significant group-time interaction, F (1,38) = 1.65, *p* = .20. However, there were statistically significant differences between the post-intervention F (1,38) = 8.93, *p* = .005, 95% CI [54.54, 75.76] and follow-up F (1,38) = 9.50, *p* = .004, 95% CI [46.88, 68.42] scores between the two groups, with the intervention group having a mean 57.65, SD = 19.58, and the control group 34.45, SD = 27.37. Finally, there were statistically significant differences between the pre-intervention and post-intervention scores of the physical activity variable within the intervention group, F(1,38) = 3.66, *p* = .035, 95% CI [pre: 30.38, 55.82, post: 54.54, 75.76], with the post-intervention scores being higher than the pre-intervention scores. There were no differences between post-intervention and follow-up scores in (*p* = .88) and no differences in the physical activity scores within the control group, F(1,38) = .74, *p* = .48. The means and SD are presented in Table 3.

### 3.6 Attitudes towards exercise & interest/enjoyment

An independent samples *t*-test was conducted to examine the differences in interest/enjoyment and attitudes towards exercise between the intervention and control groups. The analysis revealed significant differences in both variables, with the intervention group scoring significantly higher than the control group. For interest/enjoyment, the *t*-test results were t(38) = 4.247, *p* < .001, with the intervention group having a mean of 4.99, SD = 0.45 compared to the control group's mean of 4.08, SD = 0.96. Similarly, for attitudes towards exercise, the *t*-test results were t(38) = 3.477, *p* < .001, with the intervention group scoring a mean of 5.95, SD = 0.99 compared to the control group's mean of 5.37, SD = 0.74. These findings suggest that the VR exercise had a positive impact on both interest/enjoyment and attitudes towards exercise.

### 3.7 Perceived enjoyment, intended future use, usability, equipment

The descriptive statistics analysis revealed that participants expressed high levels of perceived enjoyment, intention for future use, usability, and positive perceptions of using the VR system. The maximum score for perceived enjoyment, intention for future use, and equipment was 5, while for usability it was 100. The mean and SD for each variable were as follows: perceived enjoyment had a mean of 4.93, SD = 0.12, intention for future use had a mean of 4.90, SD = 0.25, usability had a mean of 92.50, SD = 7.95, and equipment had a mean of 4.82, SD = 0.27. These results indicate that participants found the VR system highly enjoyable, intended to use it in the future, found it highly usable, and had positive perceptions of the equipment.

### 3.8 Semi-structured interview

A thematic analysis of the semi-structured interviews was conducted to explore participants’ perceptions of the intervention for both groups. The results revealed that the intervention group consistently described the VR-based exercise as highly engaging, motivating, and beneficial for their psychological well-being, while the control group emphasized the positive impact of counseling on behavior change and goal-setting. The participants’ responses are summarized in Table 4. Frequencies of the responses in the semi-structured interview are presented in Supplementary Material 8, 9.

**Table 4:** Results of the semi-structured interview.

Theme	Intervention group responses	Theme	Control group responses
Overall experience	- Innovative, highly entertaining, motivating, interesting, pleasant, comfortable, easy, accessible, and attractive.-It positively impacted their psychological well-being and they highlighted the importance of the support and guidance they received	Overall experience	- Positive, efficient, pleasant, and interesting experience, which significantly helped them achieve their goals. Their participation in the program helped them change their behavior through the goal-setting process, the guidance they received, and learning more about exercise and nutrition. - Positive sentiments towards counseling, deeming it effective, enjoyable, and instrumental in achieving personal goals. - They attributed their behavioral changes to the goal-setting process, personalized guidance, and increased knowledge about exercise and nutrition.
Counseling experience	- Enhanced consistency in the exercise regimen - Altered their perspective on exercise and nutrition - Improved their psychological state - Helped them deal with obstacles and underscored the importance of determination and concentration on their goals - Goal-setting was particularly beneficial, helping them learn how to set goals correctly, assess their needs, and use goal-setting forms effectively. This allowed them to evaluate their progress weekly and set new, more challenging goals, with advice from the exercise specialist. - Self-monitoring forms were useful for better progress evaluation and mistake monitoring. - Counseling helped them learn important information and realize the importance of psychological support and motivation for achieving their goals	Counseling experience	- The goal-setting process helped them learn to set goals correctly using the SMART method and to properly assess their needs. - Weekly evaluations and goal-setting forms facilitated progress tracking and encouraged participants to set increasingly challenging goals. - Self-monitoring forms were useful for evaluating progress, focusing on goals, and easily monitoring mistakes.
Communication, support & digital booklet	- They valued the support and guidance provided, particularly through online communication, as it helped them stay focused on their goals - The booklet was useful and interesting, providing valuable advice on managing stress and anxiety through exercise, emphasizing dedication to the program and patience, and offering tips for an active lifestyle through daily choices	Communication, support & digital booklet	- Digital booklet was deemed very useful, as they found interesting advice and tips for exercise, overeating, nutrition, and behavior change ideas. - They appreciated support and guidance, noting that online communication helped them remember their incentive and feel that there was always an expert to advise them without being time-consuming
Psychological outcomes & self-efficacy	- Increased levels of self-efficacy. - Positive changes in their mood, feeling more optimistic, motivated, self-confident, full of energy and vitality, and experiencing a sense of wellness, joy, relaxation, and reduced anxiety and stress. - Positive shift in body image, deciding to take better care of their bodies. - Increased self-confidence, reduced dropout rates, improved focus on their goals, and a feeling of being “in control.	Psychological outcomes & self-efficacy	- Heightened levels of self-efficacy. - Some of them did not experience the same increase in self-efficacy. - Being in a better mood, feeling optimistic, and having a sense of wellness - A few felt some pressure not to deviate from the program - Body image improved for half of the participants, while others always had a positive body image and wanted to take care of their bodies.
Barriers & challenges	- Challenges participants faced were time constraints and lack of energy - Communication and support combined with composure and persistence towards their goals and the digital booklet, helped them manage obstacles and challenges.	Barriers & challenges	- Challenges were lack of free time, numerous obligations, motivation, inability to access public sports facilities all day long and the need for closer monitoring and guidance by exercise and nutrition experts - Communication with the exercise specialist, optimism, persistence in their incentive, composure, self-restraint, and discipline helped them manage obstacles and challenges.
Future intentions	- Desire to continue exercising with the same or increased frequency, though time constraints remained a significant inhibitor. - Intentions to utilize behavior change techniques were high, but more likely they would use them if they had motivation and guidance, participated in a community with similar goals, or had an application that could provide feedback and encouragement	Future intentions	- They would exercise with the same or greater frequency, though time constraints were an inhibitor. - Their intention to use behavior change techniques was similar, with most participants scoring highly
VR-Based exercise	- Scored highly on its contribution to their increased levels of physical activity, noting that being able to choose the duration and intensity of their exercise reduced the stress of failure and allowed them to control the exercise program - One participant mentioned needing more challenging goals from an external source - Intended to continue exercising with the VR system and increase exercise frequency and intensity		

## 4 Discussion

The primary aim of this study was to evaluate the impact of a VR-based exercise intervention combined with SDT-based counseling on key physiological and psychological outcomes in individuals with overweight and obesity. Specifically, the study compared changes in BMI, body fat mass, basic psychological needs, attitudes towards the program, self-esteem, self-efficacy, and physical activity engagement between participants performing VR-based exercise and those engaging in traditional, self-selected exercise paired with SDT-based counseling. The secondary aim was to assess participants’ attitudes, interest, and enjoyment regarding each exercise modality, as well as the acceptance, usability, and overall enjoyment of the VR exercise platform. The results demonstrated significant improvements in physical, psychological, and behavioral factors for the intervention group compared to the control group. It was also observed increased engagement in physical activity, attitudes towards exercise, and interest/enjoyment in the intervention group compared to the control group. Finally, high scores of perceived enjoyment, intention for future use, usability and VR equipment satisfaction were reported. The results of the semi-structured interview about their experience were also encouraging.

### 4.1 Physical aspects

The study's findings indicate that the intervention significantly impacted BMI, body fat mass and hip circumference. Participants in the intervention group showed a notable reduction in BMI, body fat mass and hip circumference from pre-intervention to post-intervention measurements. While the control group also experienced decreases in these measurements, the changes were limited and not statistically significant. Many studies have examined the effects of exercise on weight loss in individuals with obesity. However, only recent studies examine the effects of VR exercise on the BMI of this population. A recent study, after an 8-week intervention with VR-based exercise, showed that the BMI was significantly decreased compared with the baseline measures and compared with traditional exercise (40). Additionally, other studies have demonstrated similar positive outcomes with innovative exercise methods. For instance, Smith et al. (60) found that combining traditional exercise with technology-based interventions, such as mobile apps and wearable devices, significantly enhanced weight loss and adherence to exercise programs compared to traditional methods alone.

### 4.2 Psychological aspects

For the BPN in general, the results revealed no statistically significant interaction between measurements and the two groups, suggesting that the overall trend in BPN in general scores did not differ significantly between the intervention and control groups across the study period. However, a closer examination showed that post-intervention, the intervention group scored significantly higher on BPN in general than the control group. Additionally, within the intervention group, there were significant increases in BPN in general scores from pre- to post-intervention, highlighting that the intervention enhanced the participants’ psychological well-being. Moreover, the analysis showed a large effect size indicating that the VR-based intervention had a substantial impact on participants’ BPN in general, and more specifically in competence. These results are consistent with previous studies that have shown the positive impact of structured physical activity on psychological well-being (61). Regarding the BPN components -Autonomy, Competence, and Relatedness- the results provided further insights. For autonomy, there were no statistically significant changes, neither between nor within groups, suggesting that the intervention did not have a significant impact on participants’ sense of autonomy. This could imply that while the intervention was beneficial overall, it did not specifically address or enhance the participants’ feelings of autonomy. The autonomy support techniques used in this study such as eliciting participants’ perspectives and sources of motivation toward participation in exercise, non-controlling language, free choice of exercise intensity and duration, experimenting, and self-initiation of the behavior may not have been sufficient to bring about changes in the satisfaction of this need, even though they were effective in previous studies. For instance, in another study, it was observed that autonomy levels were higher in self-selected exercise conditions (62). In terms of competence, the intervention group showed significant improvements post-intervention compared to the control group, and within the intervention group itself, pre- to post-intervention scores also increased. This suggests that the intervention was particularly effective in enhancing participants’ sense of competence, likely by addressing potential barriers to behavior change, setting realistic expectations, providing education on goal-setting and adjustments during counseling sessions, as well as through the use of self-monitoring forms and the educational booklet. These findings align with studies by Ntoumanis and Standage (63), who found that competence is a key predictor of motivation and engagement in physical activities. Moreover, according to Vlachopoulos & Neikou (64), the need for competence is the main predictor of exercise adherence/dropout and levels of exercise attendance and according to Banerski et al. (65) a sense of competence enhances well-being during VR exercise. The increased sense of competence may be influenced by the self-selected intensity and duration of exercise, while according to Rose and Parfitt (62), self-selected intensity and duration of exercise may positively influence the sense of autonomy and competence. For relatedness, similar to competence, the intervention group reported significantly higher post-intervention scores compared to pre-intervention, indicating that the relatedness support techniques such as expressing interest in participants’ thoughts, offering empathy, support, and motivation, allowing them to ask questions and express themselves freely, and providing positive reinforcement regardless of the outcome, were highly effective. Following our results, Reer et al. (66) revealed that VR technology can increase the satisfaction of BPN, and more specifically the needs of autonomy and competence, compared with non-VR intervention. Overall, it is clear that the intervention group showed better outcomes in two of the three components of the BPN compared to the control group. Given that the type of exercise was the only distinguishing factor between the two groups, it can be assumed that this may have contributed to the observed differences.

In this study, within the intervention group, participants exhibited significant improvements in both self-efficacy and self-esteem from pre- to post-intervention measurements. The importance of self-efficacy and self-esteem in obesity is particularly evident in the context of physical activity. Studies have shown that higher levels of self-efficacy are associated with greater participation in physical activities, which is critical for weight management and overall health (67). Additionally, improvements in self-esteem can help individuals with obesity overcome psychological barriers to engaging in physical activity, leading to more sustainable lifestyle changes (68). It was supported that regular exercise can lead to improved levels of self-efficacy, self-esteem, and body awareness (69). More specifically, according to a meta-analysis, interventions that provide feedback on participants’ performance, or vicarious experience can lead to the highest increases in self-efficacy (70). Additionally, McAuley et al. (71) demonstrated that physical activity interventions could significantly improve self-esteem, further supporting the findings of our study. Aerobic exercise has been shown to significantly enhance body self-esteem among college students, and particularly in students with overweight or obesity (72). Similar positive effects from VR exercise have been observed in both stroke patients, where self-efficacy was enhanced (73), and older adults, where self-esteem showed significant improvements (74). Additionally, college students reported higher levels of self-efficacy and enjoyment after VR biking compared to traditional biking (75, 76). Furthermore, according to the present results, there was a strong positive correlation between personal innovativeness and self-efficacy. Similarly, a study conducted on employees found that personal innovativeness was significantly correlated with participants’ self-efficacy (77). This finding means that individuals who were more innovative and open to new technology tended to have greater levels of self-efficacy. The self-esteem and self-efficacy of participants in both groups were positively affected, with the intervention group showing statistically significant differences. This indicates that VR exercise played a key role in this change, compared with traditional exercise.

There is also a small positive correlation between general mental distress and body image suggesting that individuals experiencing higher levels of health-related distress might also report concerns or dissatisfaction with their body image. As body image is closely related to psychological well-being, negative body image can affect negatively psychological well-being (78). Additionally, body image dissatisfaction is related with poor quality of life and self-esteem, depression and psychological distress (79).

### 4.3 Behavioral aspects

The results of the present study revealed changes in attitudes toward the program between the intervention and control groups post-intervention. The findings indicate that participants in the intervention group exhibited higher scores in attitudes toward the program compared to those in the control group. Moreover, the analysis showed a large effect size indicating that the VR-based intervention had a substantial impact on participants’ attitudes towards the program. These results suggest that engaging in VR exercise, as it was the only difference between the two groups, can enhance participants’ attitudes toward the program. Interestingly, while the attitudes toward the program were significantly improved within the intervention group from pre- to post-intervention, it did not have the same effect on perceived control, intentions to exercise, and subjective norms. This aligns with research by Hardeman et al. (80), who found that interventions often need to address multiple components of TPB to achieve comprehensive behavior change. Further, research by Rhodes et al. (81) supports the idea that perceived behavioral control is a crucial determinant of behavior change and may require targeted strategies to enhance an individual's confidence in their ability to perform the desired behavior. Moreover, studies such as those by Plotnikoff et al. (82) highlight the importance of social support and environmental factors in shaping behavioral intentions and actions, indicating that future interventions might benefit from incorporating these elements more robustly. Our study underscores the complexity of behavior change and the necessity for multifaceted interventions that address attitudes toward the program, and other psychological and social factors. The significant improvements in attitudes toward the program and intentions to exercise within the intervention group suggest that VR-based exercise interventions have promising potential but also highlight the need for longer-term and more comprehensive strategies to translate these changes into sustained behavior.

### 4.4 Physical activity, attitudes toward exercise and interest/enjoyment

According to our results, the intervention group demonstrated significantly higher physical activity levels post-intervention and in follow-up comparing to the control group. This suggests that engaging in VR exercise effectively increased physical activity levels immediately after the intervention and maintained these levels one month later, highlighting its lasting effect. In contrast, the control group did not exhibit any significant changes in physical activity levels throughout the study period, suggesting that the VR-based exercise option was crucial for promoting and maintaining increased physical activity. It is important to mention that the analysis showed a large effect size, which indicates a substantial difference between the physical activity levels of the intervention and control groups and not only statistical significance but also practical relevance in real-world applications. These findings are consistent with previous research indicating the effectiveness of technology-based interventions in promoting physical activity. For instance, a study by Larsen et al. (83) found that digital interventions, including VR, significantly increased physical activity levels and maintained these changes over time. Similarly, Maturo and Cunningham (84) highlighted that interactive and engaging interventions like VR can be more effective in sustaining long-term physical activity compared to traditional methods. Research suggests that VR-based exercise has the potential to enhance adherence and motivation by creating engaging and enjoyable environments (85). The ability to provide pleasurable and performance-enhancing virtual spaces makes exercise more attractive. It is also supported that while a relaxing VR environment may not promote high-intensity activity, it maximizes enjoyment, which could encourage prolonged engagement (86). Additionally, researchers highlight that active VR games can elicit various levels of physical activity intensity, with those perceived as the most enjoyable and least effortful further supporting long-term participation in physical activity (87). Moreover, there was also found a moderate positive correlation between the follow-up physical activity levels and personal innovativeness. This relationship could suggest who are more open to new technology, may also be more likely to maintain participating in VR exercise programs. There was a small positive correlation between the follow-up physical activity levels and body image. This may indicate that individuals with a more positive body image are slightly more likely to maintain or increase levels of physical activity. Following our results, Sabiston et al. (88) support that individuals with a negative perception of their body image were less likely to engage in physical activity and sports, as it was often seen as an obstacle to participation. In contrast, those with a positive body image were more inclined to be active and involved in sports.

Furthermore, results indicated that participants in the intervention group reported significantly higher levels of interest/enjoyment in exercise compared to the control group. As happened in Seo's et al. (40) study in which the levels of exercise enjoyment and immersion were significantly higher in the virtual reality exercise group compared with the other groups. This heightened interest/enjoyment could be a key factor contributing to the sustained increase in physical activity levels observed in the intervention group. According to (89), this is an important determinant for higher levels of physical activity. Moreover, research by Johnson et al. (90) suggested that interactive and immersive VR exercises could increase motivation and engagement, leading to more consistent and prolonged exercise habits among participants. There was also a moderate positive correlation between personal innovativeness and interest/enjoyment, indicating that individuals with high levels of personal innovativeness may experience more interest/enjoyment during exercise in VR environment.

Additionally, the intervention group demonstrated more positive attitudes toward exercise than the control group, further supporting the notion that engaging in VR exercise not only enhanced physical activity levels but also positively influenced participants’ perceptions and attitudes toward exercise. That is also supported by other researchers, that VR training can improve participants’ positive feelings and attitudes toward physical effort (65).

### 4.5 Exercise and counseling perceptions

According to our results, the participants of the intervention group scored highly in perceived enjoyment, intention for future use, and usability, and expressed positive perceptions of using the VR system. Following our results, VR-based physical activities can reduce anxiety and improve overall mental health, which can be particularly beneficial for individuals struggling with obesity. These findings suggest that incorporating VR technology in exercise regimens could address both physical and psychological barriers to weight loss (91).

The results from the semi-structured interviews indicated distinct differences and similarities emerged between the groups. Participants in the intervention group, who engaged in both counseling and VR exercise, consistently described the intervention as innovative and highly motivating. Significant improvements were reported in psychological well-being, consistency in their exercise regimen, and positive changes in mood and body image. The VR exercise was particularly praised for its entertainment value and ease of use, contributing to increased self-efficacy and sustained physical activity. Despite facing challenges like time constraints, the Intervention group participants expressed a strong intention to continue using the VR system and behavior change techniques, underscoring the importance of support and goal-setting in their progress. Regarding control group, which received only counseling, found the sessions positive and effective, helping them set and achieve goals related to exercise and nutrition. While most participants reported improved self-efficacy and mood, the degree of these improvements varied, with some not experiencing significant changes. Similar to the Intervention group, time constraints were a notable challenge. The digital booklet and goal-setting process were also valued in control group, aiding in progress tracking and behavioral changes. Both groups appreciated the support and guidance provided, particularly through online communication, which helped maintain focus and motivation. Overall, while both interventions were deemed effective, the addition of VR exercise in the Intervention group provided an extra layer of engagement and motivation, leading to more pronounced improvements in psychological well-being and self-efficacy compared to counseling alone in control group.

### 4.6 Limitations

Despite the encouraging results, this study has several limitations that should be mentioned. Although we conducted a power analysis to determine the appropriate sample size, and the 40 participants meet this requirement, the findings should be generalized with caution. A larger sample size could enhance the generalizability of the results. Additionally, the novelty effect of VR could have temporarily enhanced participants’ motivation and adherence to exercise. Initial enthusiasm for VR-based interventions may diminish over time as users become more familiar with this technology. Future studies could explore the long-term sustainability of VR-based exercise beyond the four-week period and assess whether the observed benefits persist over time. Investigating its effectiveness in different populations and examining the psychological mechanisms that contribute to increased enjoyment and motivation would provide deeper insights. Additionally, research should focus on the impact of VR exercise on psychological factors such as anxiety, stress, and mental well-being. Furthermore, future research could examine the cost-effectiveness of VR interventions to determine their feasibility for widespread implementation in clinical and real-world settings. Comparative studies with other emerging exercise technologies, such as AI-powered fitness platforms, could help identify the most effective tools for enhancing exercise adherence and outcomes.

### 4.7 Practical implications

Integrating VR-based exercise into obesity interventions offers a promising approach for enhancing engagement and adherence to physical activity. Fitness professionals can use VR technology to create immersive, interactive workouts tailored to individual needs and preferences. Structured VR exercise programs, such as guided virtual training sessions, can be implemented in gyms, rehabilitation centers, or even home-based settings to provide accessible and enjoyable exercise experiences. Additionally, VR can be used to simulate real-world environments that may not be feasible due to cost, safety concerns, or weather conditions, enabling individuals with obesity to engage in diverse activities within a controlled and motivating setting. Personalization features, such as adaptive difficulty levels and real-time feedback, can further enhance motivation by ensuring that workouts remain both challenging and achievable. Finally, combining VR-based exercise with behavioral counseling and SDT-based strategies may maximize long-term adherence and support sustainable lifestyle changes.

### 4.8 Conclusion

*Τ*he present study highlights the potential physical, psychological, and behavioral benefits of VR-based exercise combined with SDT-based counseling for individuals with overweight and obesity, compared to traditional exercise with SDT-based counseling. Significant improvements were observed in BMI and body fat mass, alongside enhanced psychological outcomes including increased competence, relatedness, self-efficacy, and self-esteem. These positive changes were not observed to the same extent in the control group. Additionally, VR-based exercise proved to be effective in sustaining physical activity levels, likely due to high levels of enjoyment, positive attitudes toward exercise, great psychological needs satisfaction, usability, and satisfaction with the VR equipment.

## Data Availability

The raw data supporting the conclusions of this article will be made available by the authors, without undue reservation.
