# Supplementary material for: Effectiveness and acceptance of virtual reality vs. traditional exercise in obese adults: a pilot randomized trial
Source: Front Sports Act Living. 2025 Mar 19;7:1520068. doi: 10.3389/fspor.2025.1520068 (PMC11962008; doi:10.3389/fspor.2025.1520068)
Supplement: Supplementary file 1 [file Datasheet1.pdf]

Πάμε να θέσουμε στόχους με βάση το σύστημα **SMART**. Θα μας βοηθήσει να θέσουμε σαφείς , μετρήσιμους, εφικτούς, ρεαλιστικούς και χρονικά προσδιορισμένους στόχους. Συνεπώς, πιο αποτελεσματικούς.

Συγκεκριμένος :

Ο στόχος πρέπει να είναι σαφής και προσδιορισμένος. Πρέπει να είναι ξεκάθαρο τι ακριβώς θέλουμε να επιτύχουμε.

Μετρήσιμος :

Πρέπει να είναι δυνατό να μετρήσουμε την πρόοδο προς την επίτευξη του στόχου. Αυτό συμβάλλει στην παρακολούθηση και αξιολόγηση των αποτελεσμάτων.

Εφικτός :

Ο στόχος πρέπει να είναι ρεαλιστικός και εφικτός. Πρέπει να είναι κάτι που μπορούμε να πραγματοποιήσουμε με τους διαθέσιμους πόρους.

Ρεαλιστικός :

Πρέπει να είναι αντίστοιχος με τους στόχους και τις δυνατότητές μας. Πρέπει να είναι κάτι που είναι εφικτό με την προσπάθειά μας.

Χρονικά Προσδιορισμένος :

Πρέπει να έχει καθορισμένο χρονικό πλαίσιο. Πρέπει να ξέρουμε πότε πρόκειται να επιτύχουμε τον στόχο.

Παράδειγμα:

"Θέλω να χάσω 5 κιλά (συγκεκριμένος), μετρήσιμα μέσω της ζυγαριάς, τους επόμενους 2 μήνες (χρονικά προσδιορισμένος), και θα το πετύχω με την καθημερινή άσκηση και υγιεινή διατροφή (εφικτός και ρεαλιστικός)."

Θέσε έναν στόχο για φυσική δραστηριότητα/άσκηση, έναν στόχο για διατροφή και έναν για νερό για τις επόμενες 2 εβδομάδες.

**Καθορισμός στόχων SMART**						
**Ημ/νια**	**Στόχος**	**Πως θα μετρηθεί;**	**Πότε**	**Τι θέλω να πετύχω με αυτό;**	**Επιβράβευση στον εαυτό μου**	**Πραγματο-ποιήθηκε;**

Σχέδιο πρόθεσης εφαρμογής

Παράδειγμα

"Την επόμενη εβδομάδα (πότε), στις 7:00 π.μ. (πώς), θα πηγαίνω στο γυμναστήριο και θα ασκούμαι για 30 λεπτά τρεις φορές την εβδομάδα (τι) για να βελτιώσω τη φυσική μου κατάσταση."

Ημερομηνία :

Δήλωση :

Για να καταφέρω να (στόχος)…………………………………………………………………………………………..,

θα (πράξη/δραστηριότητα) ………………………………………………………………………………………………

(πότε) …….,

(πώς) ………………… ……………………………………………………………………………………………………

Ημερομηνία :

Δήλωση :

Για να καταφέρω να (στόχος)…………………………………………………………………………………………………..,

θα (πράξη/δραστηριότητα) ………………………………………………………………………..…………………………

(πότε) ……,

(πώς) …………………..………………………………………………………………………………………..………………….

Ημερομηνία :

Δήλωση :

Για να καταφέρω να (στόχος)…………………………………………………………………………………………………..,

θα (πράξη/δραστηριότητα) ………………………………………………………………………..…………………………

(πότε) ……,

(πώς) …………………... ………………………………………………………………………………………..………………….
