# Supplementary material for: Effectiveness and acceptance of virtual reality vs. traditional exercise in obese adults: a pilot randomized trial
Source: Front Sports Act Living. 2025 Mar 19;7:1520068. doi: 10.3389/fspor.2025.1520068 (PMC11962008; doi:10.3389/fspor.2025.1520068)
Supplement: Supplementary file 2 [file Datasheet2.pdf]

Φόρμα καθημερινής αυτό-παρακολούθησης
(συμπλήρωσε για 7 συνεχόμενες ημέρες)

	**Ημέρα 1η**	**Ημέρα 2η**	**Ημέρα 3η**	**Ημέρα 4η**	**Ημέρα 5η**	**Ημέρα 6η**	**Ημέρα 7η**
Πόσα βήματα έκανες σήμερα; (δες το κινητό σου)							
Πόσο νερό ήπιες σήμερα; (σε ποτήρια)							
Πόσα λεπτά φυσικής δραστηριότητας έκανες σήμερα; (π.χ. περπάτημα, ποδήλατο κλπ.)							
Πόσα φρούτα έφαγες σήμερα;							
Πόσα λαχανικά έφαγες σήμερα;							
Πόσα τρόφιμα ή ποτά κατανάλωσες σήμερα που είχαν μεγάλη ποσότητα ζάχαρης; (αναψυκτικά, γλυκά κλπ)							
