# Supplementary material for: Effectiveness and acceptance of virtual reality vs. traditional exercise in obese adults: a pilot randomized trial
Source: Front Sports Act Living. 2025 Mar 19;7:1520068. doi: 10.3389/fspor.2025.1520068 (PMC11962008; doi:10.3389/fspor.2025.1520068)
Supplement: Supplementary file 3 [file Image1.pdf]

ΕΞΕΡΕΥΝΗΣΕ ΤΗΝ ΑΣΚΗΣΗ ΣΕ ΠΕΡΙΒΑΛΛΟΝ ΕΙΚΟΝΙΚΗΣ ΠΡΑΓΜΑΤΙΚΟΤΗΤΑΣ

Virtual Reality Exercise Program

Είσαι 18-65 ετών;
Έχεις βάρος πάνω από το ενδεικνυόμενο;

ΕΝΑΣ ΜΗΝΑΣ ΔΩΡΕΑΝ!

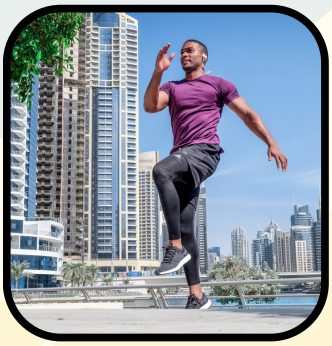

ΕΝΤΑΞΟΥ ΣΕ ΠΡΟΓΡΑΜΜΑ ΑΣΚΗΣΗΣ

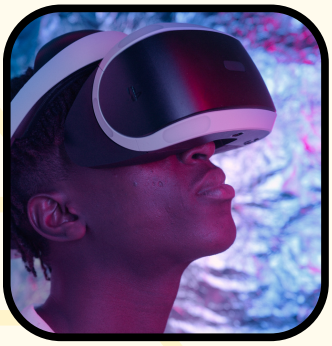

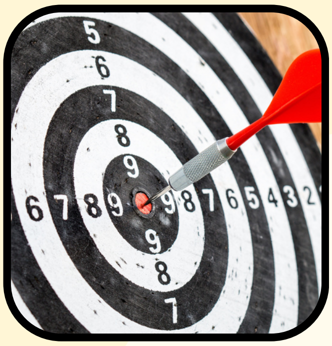

ΣΥΜΒΟΥΛΕΥΤΙΚΗ ΥΠΟΣΤΗΡΙΞΗ

ΚΑΝΕ ΑΣΚΗΣΗ ΣΕ ΕΙΚΟΝΙΚΟ ΠΕΡΙΒΑΛΛΟΝ

Συμπλήρωσε τη φόρμα για να λάβεις μέρος στο πιλοτικό πρόγραμμα άσκησης σε περιβάλλον εικονικής πραγματικότητας σε συνεργασία με το Πανεπιστήμιο Θεσσαλίας.

ΔΗΛΩΣΕ ΣΥΜΜΕΤΟΧΗ

ΣΚΑΝΑΡΕ ΚΑΙ ΣΥΜΠΛΗΡΩΣΕ

Ή κάλεσε στο [redacted] για περισσότερες πληροφορίες

ΠΑΝΕΠΙΣΤΗΜΙΟ ΘΕΣΣΑΛΙΑΣ
ΕΤΟΣ ΙΔΡΥΣΗΣ 1984
