# Supplementary material for: Effectiveness and acceptance of virtual reality vs. traditional exercise in obese adults: a pilot randomized trial
Source: Front Sports Act Living. 2025 Mar 19;7:1520068. doi: 10.3389/fspor.2025.1520068 (PMC11962008; doi:10.3389/fspor.2025.1520068)
Supplement: Supplementary file 4 [file Image2.pdf]

Εργαστήριο Ψυχολογίας της Άσκησης
& Ποιότητας Ζωής

ΤΕΦΑΑ ΠΘ

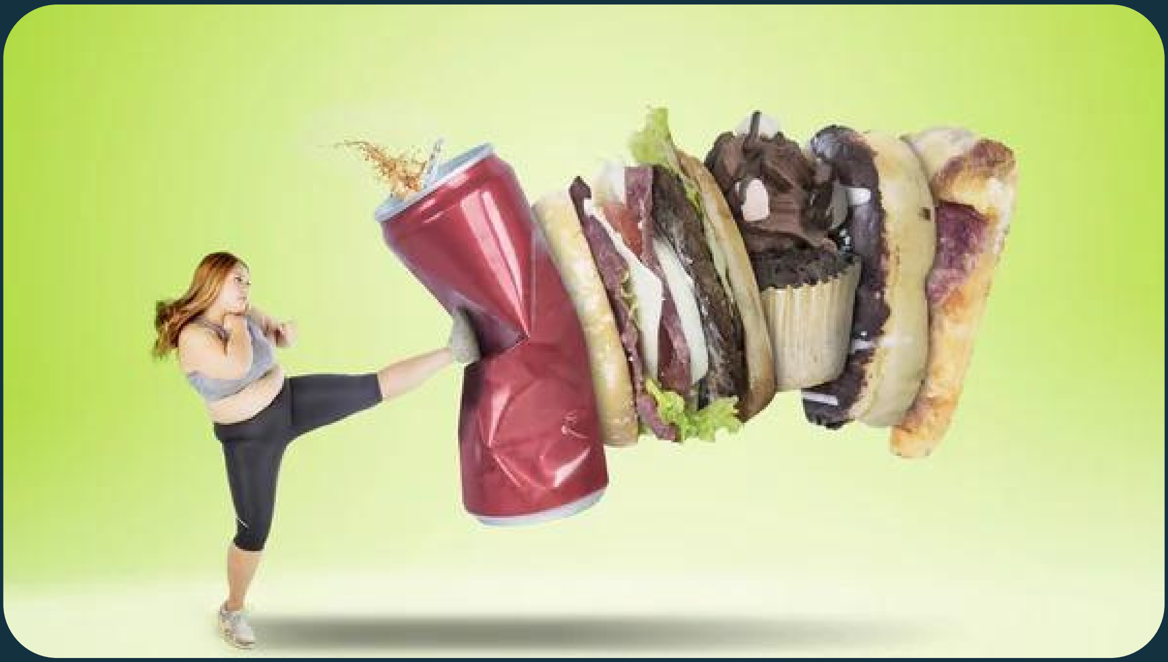

ΚΑΝΕ ΚΑΤΙ ΣΗΜΕΡΑ ΩΣΤΕ Ο ΜΕΛΛΟΝΤΙΚΟΣ ΕΑΥΤΟΣ ΣΟΥ ΝΑ Σ' ΕΥΧΑΡΙΣΤΕΙ ΓΙΑ ΑΥΤΟ.

Μικρές συμβουλές που θα σε βοηθήσουν να πετύχεις τους στόχους σου

http://psych.pe.uth.gr/index.php/el/

Πόσο δύσκολο είναι να επιτύχει κάνεις τον έλεγχο του βάρους του;

Είναι περισσότερο θέμα κατάλληλης διατροφής, κατάλληλης άσκησης, εσωτερικής προσπάθειας και αυτοελέγχου ή κατάλληλης εκπαίδευσης;

Στο πλήθος των μεθόδων ελέγχου του βάρους που προτείνονται, πολυδιαφημιζόμενων ή μη, ποιοι είναι οι χρυσοί κανόνες που θα πρέπει να ακολουθεί κάνεις;

Στο φυλλάδιο αυτό, αναπτύσσονται οι δυσκολίες στον έλεγχο των συμπεριφορών που σχετίζονται με το βάρος και την παχυσαρκία, ο ρόλος της άσκησης και της διατροφής, όπως επίσης και η σχέση του στρες με τον έλεγχο του βάρους. Περιγράφεται πλήθος ιδεών και τεχνικών, για τον έλεγχο του βάρους, όπως επίσης και το ιδιαίτερο θέμα των διατροφικών διαταραχών.

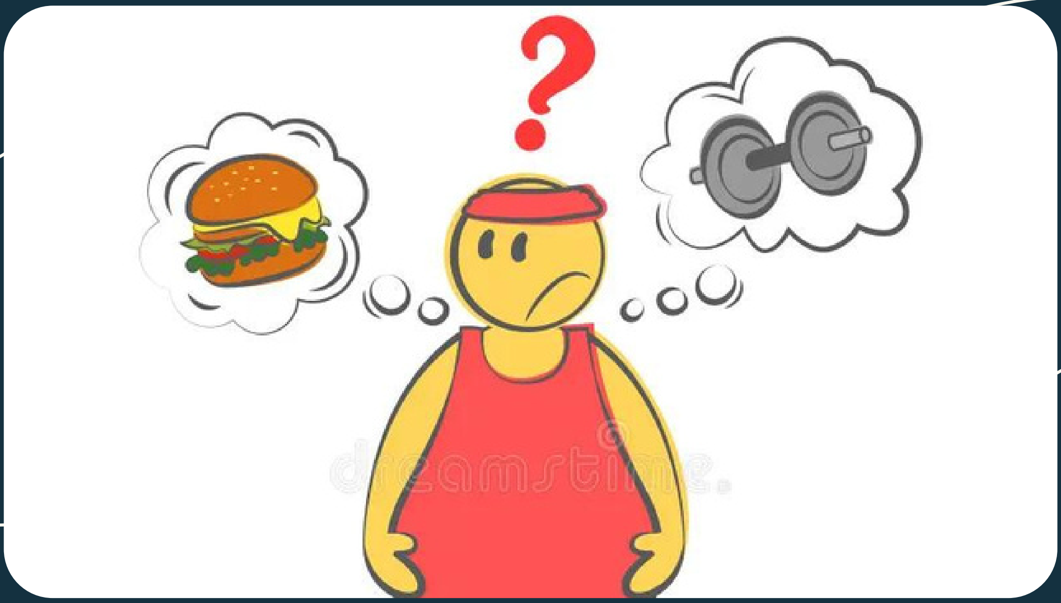

ΕΙΣΑΓΩΓΗ

Ο Ιπποκράτης πολλούς αιώνες πριν είπε ότι ο άνθρωπος δεν μπορεί να είναι υγιής τρώγοντας μόνο, αν δε γυμνάζεται επίσης. Η αναζήτηση της ισορροπίας ανάμεσα στην κίνηση του σώματος και την διατροφή είναι ένα από τα μικρά μυστικά της υγείας των ατόμων και η διαταραχή αυτής της ισορροπίας εκφράζεται με πολλούς τρόπους.

Η παχυσαρκία είναι μία πολυπαραγοντική νόσος που χαρακτηρίζεται από διαταραχές στο ενδοκρινικό σύστημα και στο μεταβολισμό, αλλά και διαταραχές στην συμπεριφορά του ατόμου. Θεωρείται ως μία χρόνια και υποτροπιάζουσα νόσος και αποτελεί μείζον πρόβλημα της δημόσιας υγείας. Η παχυσαρκία συνδέεται με την εμφάνιση άλλων χρόνιων νοσημάτων, όπως είναι η αρτηριακή υπέρταση, ο σακχαρώδης διαβήτης τύπου 2, οι καρδιακές νόσοι και τα αγγειακά εγκεφαλικά επεισόδια, η δυσλιπιδαιμία, το σύνδρομο άπνοιας ύπνου, το άσθμα και οι ορμονοεξαρτώμενοι καρκίνοι.

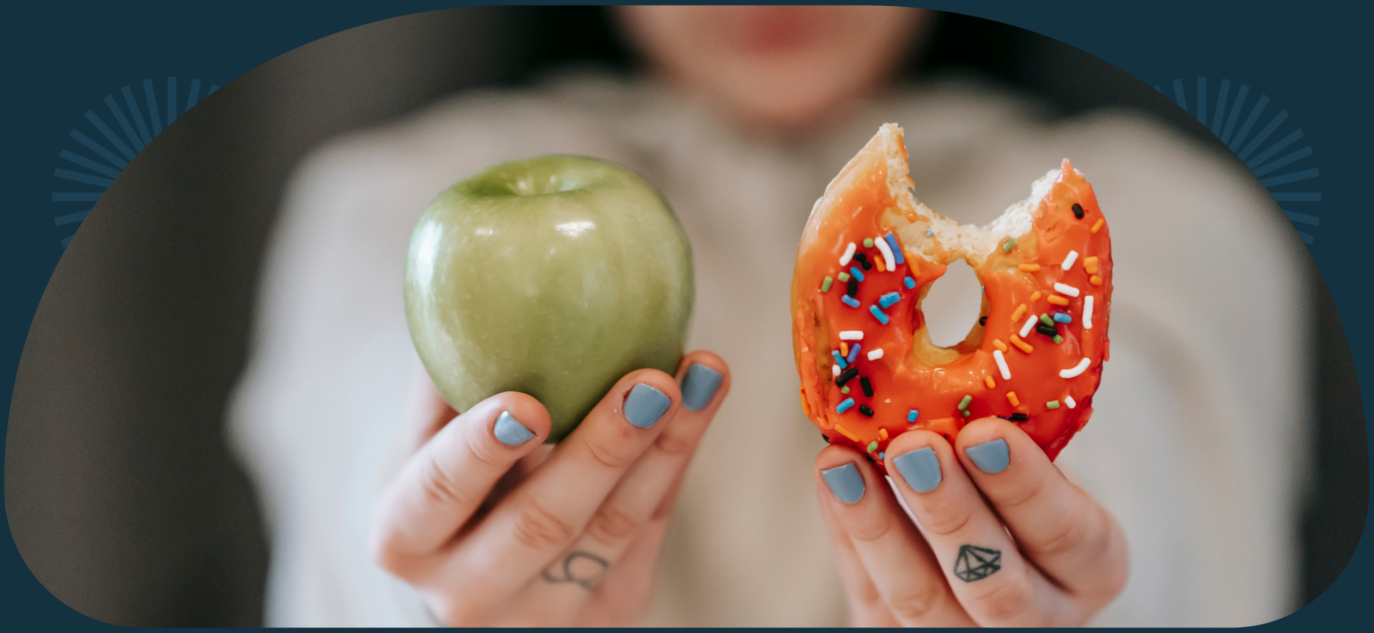

Πόση άσκηση είναι επαρκής για την απώλεια σωματικού βάρους και τη διατήρησή του;

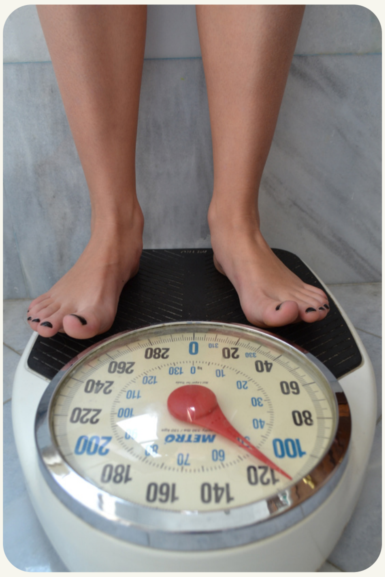

Σε παχύσαρκα άτομα η αύξηση της φυσικής δραστηριότητας, μπορεί να μειώσει τον κίνδυνο εμφάνισης καρδιαγγειακών νοσημάτων ακόμη και χωρίς απώλεια σωματικού βάρους. Υ

Η υιοθέτηση υποθερμιδικής δίαιτας (με μείωση 1000-1500 kcal/ημέρα) σε συνδυασμό με συμμετοχή σε προγράμματα άσκησης, προκαλούν μεγαλύτερη απώλεια σωματικού βάρους και λίπους. Ο συνδυασμός δίαιτας και άσκησης συμβάλλει στη διατήρηση του επιθυμητού σωματικού βάρους για μεγαλύτερο χρονικό διάστημα. Η απώλεια σωματικού βάρους και λίπους, που επιτυγχάνεται με την άσκηση, είναι ανάλογη της ενεργειακής δαπάνης (δηλαδή του συνολικού όγκου της άσκησης που εκτελείται).

Πίνακας 3. Βασικές οδηγίες για τη βελτίωση της αερόβιας ικανότητας σε παχύσαρκα άτομα [142, 155-158].

Στοιχεία επιβάρυνσης

Συχνότητα
- 5 φορές/εβδομάδα (τουλάχιστον 150-200 min/εβδομάδα).

Ένταση
- 60-85% της μέγιστης καρδιακής συχνότητας.

Διάρκεια
- 45-60 min/ημέρα.

Μέθοδος προπόνησης
- Συνεχόμενη.
- Διαλειμματική: με περιόδους που μπορούν να πραγματοποιηθούν και σε διαφορετικά χρονικά διαστήματα κατά τη διάρκεια της ημέρας (π.χ. 6 σετ x 5 min ή 3 σετ x 10 min ή 2 σετ x 15 min).

Προπονητικά Περιεχόμενα

Δραστηριότητες που γυμνάζουν μεγάλες μυϊκές ομάδες, όπως περπάτημα, άσκηση σε ελλειπτικό μηχάνημα, ποδηλασία, κολύμβηση κ.α. Δίνεται έμφαση σε δραστηριότητες που δεν περιλαμβάνουν μεταφορά του σωματικού βάρους και δεν προκαλούν μεγάλους κραδασμούς.

Προοδευτική αύξηση της επιβάρυνσης

Καθ' όλη τη διάρκεια του προγράμματος παρέμβασης πραγματοποιείται σταδιακή αύξηση της επιβάρυνσης. Η αλλαγή των στοιχείων της επιβάρυνσης προτείνεται να ακολουθεί την παρακάτω σειρά: αύξηση της συχνότητας άσκησης (από 3 σε 4-5 φορές την εβδομάδα ή και καθημερινά), μετά της διάρκειας και στη συνέχεια της έντασης της άσκησης (από 60 σε 70-85% της μέγιστης καρδιακής συχνότητας).

Πως θα βρω τη μέγιστη καρδιακή συχνότητά μου (HRmax);

Αφαίρεσε από 220 την ηλικία σου και στη συνέχεια πολλαπλασίασε τον αριθμό που βρήκες με το 0,6 (αν θέλεις το 60%) ή το 0,70 (αν θέλεις το 70%) κοκ.

Παράδειγμα : Ένα άτομο 40 ετών θέλει να κάνει αερόβια άσκηση στο 80% της μέγιστης καρδιακής συχνότητάς του.

220-40=180 , 180 x 0,8 = 144

Το άτομο θα κάνει άσκηση με 144 παλμούς ανά λεπτό, μπορεί να κυμαίνεται από 140-150 παλμούς περίπου.

Στόχοι συμμετοχής στην άσκηση

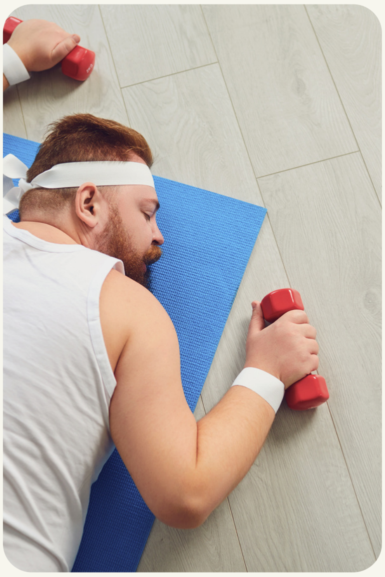

Ο κύριος στόχος της συμμετοχής σε συστηματική άσκηση είναι η μείωση του λιπώδους ιστού, και κατ΄ επέκταση η μείωση του σωματικού βάρους.
Η aερόβια άσκηση βελτιώνει την λειτουργική ικανότητα και συμβάλλει στη μείωση του ποσοστού σωματικού λίπους.
Η άσκηση με αντιστάσεις (βάρη) συμβάλλει στην αύξηση της μυϊκής μάζας, την πρόληψη των πτώσεων και την αύξηση του βασικού μεταβολισμού.
Τα άτομα με παχυσαρκία θα πρέπει προοδευτικά, να αυξήσουν την ενασχόλησή τους με την άσκηση. Επιθυμητό είναι η συνολική ενασχόληση με την άσκηση να υπερβαίνει τα 280 λεπτά την εβδομάδα. Ο ρόλος της άσκησης είναι σημαντικός στην διατήρηση του σωματικού βάρους, μετά από την απώλεια των περιττών κιλών.

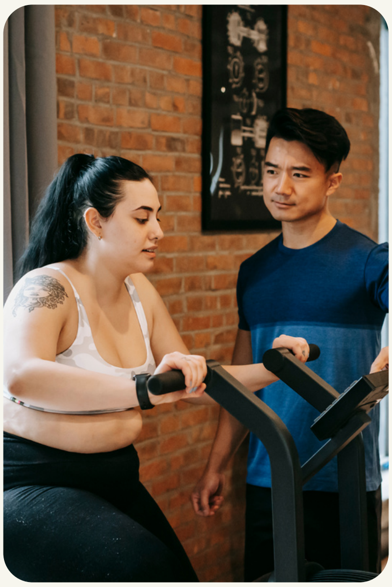

- Μείωση σωματικού λίπους
- Αύξηση κατανάλωσης ενέργειας (σωστή κατανομή της έντασης και της διάρκειας της άσκησης)
- Αύξηση μυικής μάζας και βασικού μεταβολισμού
- Η ένταση της άσκησης ειδικά στα αρχικά στάδια να είναι χαμηλή έως μέτρια και οι αθλούμενοι να είναι ιδιαίτερα προσεκτικοί στην επιλογή της φυσικής δραστηριότητας
- Να επιλέγονται δραστηριότητες που δεν περιλαμβάνουν μεταφορά του σωματικού βάρους για παράδειγμα ποδήλατο, άσκηση σε ελλειπτικό μηχάνημα, ασκήσεις στο νερό κλπ
- Κατά την προπονητική διαδικασία μπορεί να χρειάζονται συχνές προσαρμογές την συχνότητα και την διάρκεια της άσκησης

Ενδεικτικό πρόγραμμα για βελτίωση της αντοχής στη δύναμη

Προθέρμανση (10 min)

- Χαμηλή έως μέτριας έντασης αερόβια δραστηριότητα.
- Διατατικές ασκήσεις (δυναμικές και στατικές).

- **Στόχος:** Αντοχή στη δύναμη.
- **Επαναλήψεις:** 15 επαναλήψεις/σειρά (σετ).
- **Σειρές (σετ):** 3 σειρές/άσκηση.
- **Διάλειμμα:** 2 min/σειρά, 2 min/άσκηση.
- **Μορφή οργάνωσης της προπόνησης:** Κυκλική προπόνηση.
- **Προπονητικά περιεχόμενα:** 7 ασκήσεις με το βάρος του σώματος ή με αντιστάσεις (αλτήρες, λάστιχο).

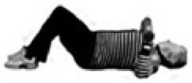

Άσκηση 1
Άρσεις κορμού από ύπτια κατάκλιση με τα γόνατα σε κάμψη (κοιλιακοί)

Άσκηση 2
Πιέσεις στήθους με αλτήρες

Άσκηση 3
Εκτάσεις γονάτων με λάστιχο

Άσκηση 4
Εναλλάξ άρσεις ποδιών και χεριών από την πρηνή κατάκλιση

Άσκηση 5
Πρόσθιες άρσεις χεριών με αλτήρες

Άσκηση 6
Άρση από καρέκλα

Άσκηση 7
Εκτάσεις τρικεφάλων με αλτήρες από καθιστή θέση

x 3 σειρές

Αποκατάσταση (10 min)

- Διατατικές ασκήσεις (στατικές μυϊκές διατάσεις).
- Αναπνευστικές ασκήσεις χαλάρωσης.

Σχήμα 7. Ενδεικτικό πρόγραμμα για τη βελτίωση της αντοχής στη δύναμη παχύσαρκων ατόμων.

Ενδεικτικό πρόγραμμα για βελτίωση της αερόβιας ικανότητας

Ενδεικτικά προγράμματα για τη βελτίωση της αερόβιας ικανότητας

Προθέρμανση (10 min)

- Tai chi.
- Διατατικές ασκήσεις (δυναμικές και στατικές ασκήσεις).

Πρόγραμμα 1

- **Μέθοδος προπόνησης:** Διαλειμματική.
- **Διάρκεια:** 50min (5σετ x 10min, με 3min διάλειμμα/σετ).
- **Ένταση:** 70-75% HR_{max}.
- **Δραστηριότητα:** Στο $1^{ο}$ και $2^{ο}$ σετ περπάτημα (σε εργοδιάδρομο), στο $3^{ο}$ και $4^{ο}$ σετ άσκηση με ελλειπτικό μηχάνημα (με κίνηση στα άνω και κάτω άκρα) και στο $5^{ο}$ σετ ποδήλατο.

Πρόγραμμα 2

- **Μέθοδος προπόνησης:** Συνεχόμενη.
- **Διάρκεια:** 50 min
- **Ένταση:** 60-70% HR_{max}.
- **Δραστηριότητα:** Αερόβιος χορός στο νερό (aqua aerobic).
  - Ασκήσεις χαμηλής κρούσης.
  - Κίνηση των χεριών τόσο κάτω όσο και πάνω από το επίπεδο των ώμων.
  - Δίνεται έμφαση στο εύρος κίνησης.

Αποκατάσταση (10 min)

- Διατατικές ασκήσεις (στατικές μυϊκές διατάσεις).
- Αναπνευστικές ασκήσεις.

Σχήμα 5. Ενδεικτικά προγράμματα για τη βελτίωση της αερόβιας ικανότητας παχύσαρκων ατόμων.

HR_{max}: μέγιστη καρδιακή συχνότητα.

Ενδεικτικό πρόγραμμα για βελτίωση της δύναμης με βάρη

Προθέρμανση (10 min)

- Χαμηλής έως μέτριας έντασης αερόβια δραστηριότητα.
- Διατατικές ασκήσεις (δυναμικές και στατικές).

- **Στόχος:** Μέγιστη δύναμη με μυϊκή υπερτροφία.
- **Επαναλήψεις:** 10 σε κάθε σειρά (σετ).
- **Σειρές (σετ):** 3.
- **Διάλειμμα:** 2 min/σειρά, 3 min/άσκηση.
- **Μορφή οργάνωσης της προπόνησης:** Προπόνηση σε σταθμούς.
- **Προπονητικά περιεχόμενα:** 6 ασκήσεις με μηχανήματα δύναμης.

Άσκηση 1 Πιέσεις ποδιών → **Άσκηση 2** Κωπηλατική από εδραία θέση → **Άσκηση 3** Εκτάσεις αγκώνων από καθιστή θέση → **Άσκηση 4** Peck - Deck → **Άσκηση 5** Κάμψεις γονάτων → **Άσκηση 6** Πλάγιες άρσεις χεριών από καθιστή θέση

Αποκατάσταση (10 min)

- Διατατικές ασκήσεις (στατικές μυϊκές διατάσεις).
- Αναπνευστικές ασκήσεις.

Σχήμα 6. Ενδεικτικό πρόγραμμα για τη βελτίωση της μέγιστης δύναμης (με μυϊκή υπερτροφία) παχύσαρκων ατόμων με χρήση μηχανημάτων αντίστασης (βάρη).

Οι παρακάτω πίνακες προσφέρουν χρήσιμες οδηγίες για την προπόνηση δύναμης και κινητικότητας

Πίνακας 4. Βασικές οδηγίες για την προπόνηση δύναμης σε παχύσαρκα άτομα [164-168].

Στοιχεία επιβάρυνσης

Συχνότητα

- 2-3φορές/εβδομάδα.

Ένταση

- Για βελτίωση της μυϊκής δύναμης: μέτρια έως υψηλή (60-80% 1ΜΕ).
- Για βελτίωση της μυϊκής αντοχής: χαμηλή έως μέτρια (10-50% 1ΜΕ).

Επαναλήψεις

- Για βελτίωση της μυϊκής δύναμης: 8-12.
- Για βελτίωση της μυϊκής αντοχής: 15-20.

Ποσότητα

- 6-8 ασκήσεις/προπονητική μονάδα.
- 2-4 σειρές (σετ) ανά άσκηση.
- 3-5 min διάλειμμα μεταξύ των σειρών.

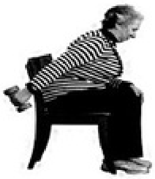

Μορφή οργάνωσης της προπόνησης

- Προπόνηση σε σταθμούς.
- Κυκλική προπόνηση.

Προπονητικά Περιεχόμενα

- Ασκήσεις με το βάρος του σώματος (κοιλιακούς, κάμψεις, ραχιαίους κ.α.).
- Ασκήσεις με βοηθητικά όργανα (μπάλες ισορροπίας, ιατρικές μπάλες, μπαλάκια κ.α.).
- Ασκήσεις με μηχανήματα δύναμης.

1ΜΕ: 1 Μέγιστη επανάληψη.

Πίνακας 5. Βασικές οδηγίες για την προπόνηση κινητικότητας σε παχύσαρκα άτομα [165, 169].

Στοιχεία επιβάρυνσης

Συχνότητα

- 3 φορές/εβδομάδα.

Ένταση

- Η διάταση πρέπει να εκτελείται στο πλήρες εύρος κίνησης της άρθρωσης (χωρίς να προκαλείται πόνος).

Διάρκεια

- Στατικές μυϊκές διατάσεις: 10-20 s/επανάληψη.
- Διατάσεις ιδιοδέκτριας νευρομυϊκής διευκόλυνσης (PNF): 3-6 s ισομετρική σύσπαση και στη συνέχεια 10-15 s στατική διάταση.
- Δυναμικές μυϊκές διατάσεις: 10-20 επαναλήψεις/σειρά.

Σειρές (σετ) - Επαναλήψεις

2-4 σειρές - επαναλήψεις/άσκηση.

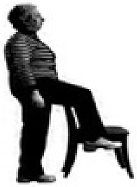

Μέθοδοι μυϊκών διατάσεων

Στατικές μυϊκές διατάσεις.
Δυναμικές μυϊκές διατάσεις.
Ιδιοδέκτρια νευρομυϊκή διευκόλυνση (PNF) (χρησιμοποιείται σε πιο προχωρημένου επιπέδου ασκούμενους).

Προπονητικά Περιεχόμενα

Διατατικές ασκήσεις με το βάρος του σώματος.
Διατατικές ασκήσεις με βοηθητικά όργανα (λάστιχα, μπάλες ισορροπίας, κτλ.).
Μορφές άσκησης, όπως yoga, tai chi κτλ.

«Δεν χάνω κιλά» , «έχει κολλήσει η ζυγαριά», «έπρεπε να έχω χάσει κάποια γραμμάρια/κιλά αυτή την εβδομάδα», «δεν έχω την απώλεια που υπολόγιζα»

Η έλλειψη ακρίβειας στην πρόβλεψη απώλειας του σωματικού βάρους μετά την εφαρμογή ενός προγράμματος άσκησης, απογοητεύει αρκετούς ασκούμενους. Θα πρέπει να γίνει κατανοητό από τους ασκούμενους, ότι ο ρυθμός προσαρμογής ερεθίσματα άσκησης μπορεί να διαφέρει σημαντικά από άτομο σε άτομο. Το γεγονός αυτό, οφείλεται σε γενετικούς παράγοντες, στην κατάσταση της υγείας του ατόμου και στην λειτουργική του ικανότητα. Ορισμένα άτομα ανταποκρίνονται ταχύτερα στα προπονητικά ερεθίσματα, ενώ άλλα άτομα καθυστερούν. Υπάρχει δηλαδή εξατομίκευση την απώλεια του σωματικού βάρους που μπορεί να επιτευχθεί με την άσκηση.

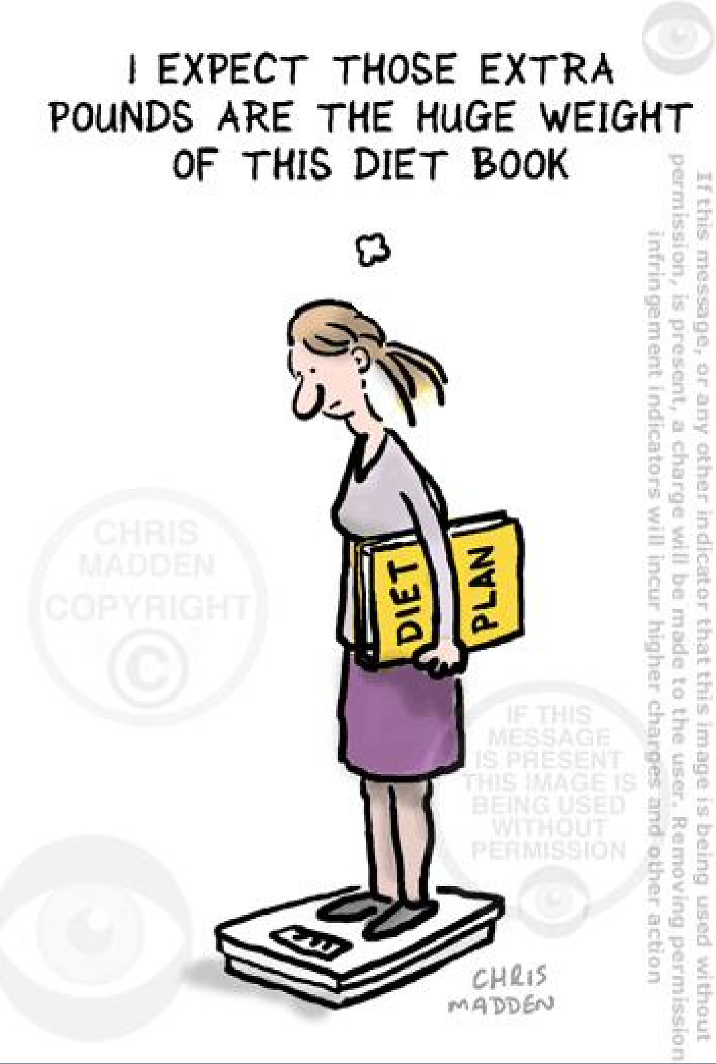

Συμβουλές για μια Υγιεινή και Ισορροπημένη Διατροφή

Ο Παγκόσμιος Οργανισμός Υγείας (ΠΟΥ) και άλλοι επιστημονικοί φορείς παρέχουν βασικές κατευθυντήριες οδηγίες για τη διατροφή παχύσαρκων ατόμων με σκοπό την απώλεια βάρους και την αύξηση της γενικής υγείας. Παρακάτω, παρουσιάζονται βασικές συστάσεις βασισμένες στην επιστημονική βιβλιογραφία και τις κατευθυντήριες οδηγίες του ΠΟΥ:

Μείωση των Θερμίδων: Η απώλεια βάρους απαιτεί την κατανάλωση λιγότερων θερμίδων από αυτές που δαπανώνται. Αυτό επιτυγχάνεται με τη μείωση του συνολικού ποσού τροφής και την επιλογή τροφίμων με χαμηλή πυκνότητα θερμίδων.

Επιλογή Υγιεινών Τροφίμων: Προτίμηση τροφίμων υψηλής θρεπτικής αξίας, όπως φρούτα, λαχανικά, πηγές πρωτεΐνης όπως το κοτόπουλο και το ψάρι, δημητριακά ολικής άλεσης και υγιεινά λίπη όπως το αβοκάντο και το ελαιόλαδο.

Περιορισμός των Ανεπιθύμητων Συστατικών: Περιορισμός στην κατανάλωση κορεσμένων λιπαρών, απλών σακχάρων και αλατιού. Αποφυγή αναψυκτικών πλούσια σε σάκχαρα και τα επεξεργασμένων τροφίμων.

Ελέγχος της ποσότητας του φαγητού: Προσοχή στο μέγεθος των μεριδών φαγητού. Μικρότερες μερίδες μπορούν να βοηθήσουν στον έλεγχο της πείνας και της κατανάλωσης θερμίδων.

Πρωινό: Αποφυγή παράλειψης του πρωινού. Ένα υγιές πρωινό μπορεί να βοηθήσει στον έλεγχο της πείνας κατά τη διάρκεια της ημέρας.

Νερό: Κατανάλωση αρκετής ποσότητας νερού κατά τη διάρκεια της ημέρας, καθώς ορισμένες φορές η αίσθηση της πείνας μπορεί να συγχέεται με τη δίψα.

Στήριξη και Συμβουλές: Ένας επαγγελματίας υγείας ή διαιτολόγο που μπορεί να δημιουργήσει ένα εξατομικευμένο σχέδιο διατροφής και να παρέχει χρήσιμες συμβουλές και στήριξη.

Η απώλεια βάρους είναι μια μακροπρόθεσμη πρόκληση. Οι παραπάνω συστάσεις είναι γενικές κατευθυντήριες οδηγίες και μπορεί να προσαρμοστούν στις ατομικές ανάγκες και προτιμήσεις.

Οι 8 κορυφαίες συμβουλές βασισμένες στη συνήθεια για τον έλεγχο του βάρους

**Κράτησε τη ρουτίνα των γευμάτων σου**	
Προσπάθησε να τρως τις ίδιες ώρες κάθε μέρα, ακόμη κι αν είναι 2 ή 5 φορές την ημέρα	Αυτή η συμβουλή βοηθά στην ανάπτυξη της συνήθειας
**Κινήσου**	
Προσπάθησε να περπατήσεις 10.000 βήματα (περίπου 60-90 λεπτά φυσική δραστηριότητα μέτριας έντασης) κάθε μέρα. Μπορείς να χρησιμοποιήσεις εφαρμογές που μετρούν τα βήματα για να σε βοηθήσουν.	Εκτιμώμενο ημερήσιο έλλειμμα 100-200 θερμίδων
**Ετοίμασε υγιεινά σνακ**	
Διάλεξε μια υγιεινή επιλογή για σνακ όπως φρούτα, γιαούρτι με χαμηλές θερμίδες αντί για σοκολάτα ή πατατάκια κλπ.	Υπολογίζεται να αποφύγεις τουλάχιστον 150 θερμίδες
**Διάβασε τις ετικέτες**	
Πρόσεξε τις ετικέτες των τροφίμων που αναφέρουν τα συστατικά. Έλεγξε την περιεκτικότητά τους σε λιπαρά και σάκχαρα όταν αγοράζεις τρόφιμα.	Αυτή η συμβουλή βοηθά να κάνεις επιλογές με σωστή πληροφόρηση σχετικά με το φαγητό σου

Οι 8 κορυφαίες συμβουλές βασισμένες στη συνήθεια για τον έλεγχο του βάρους

**Να αποφεύγεις την καθιστική συμπεριφορά**	
Κάνε διαλείμματα από παρατεταμένη καθιστική θέση. Κάθε 1 ώρα σήκω για 10 λεπτά.	Εκτιμώμενο ημερήσιο έλλειμμα 100 θερμίδων
**Πρόσεξε τα ποτά σου**	
Διάλεξε να πίνεις νερό ή ποτά χωρίς ζάχαρη. Ο φυσικός χυμός περιέχει φυσικά σάκχαρα (προτείνεται 1 ποτήρι την ημέρα). Τα αλκοολούχα ποτά είναι πλούσια σε θερμίδες, περιόρισέ τα (1 για τις γυναίκες και 2 για τους άνδρες)	Εκτιμώμενο ημερήσιο έλλειμμα 150 θερμίδων
**Συγκεντρώσου στο φαγητό σου**	
Φάε ήρεμα, μη βλέπεις τηλεόραση κατά τη διάρκεια του φαγητού. Εάν μπορείς, φάε στο τραπέζι.	Αυτή η συμβουλή θα σε βοηθήσει να αφοσιωθείς στις υγιεινές σου συνήθειες και να αποφύγεις ανθυγιεινές συμπεριφορές
**Να μην ξεχνάς το «5 την ημέρα»**	
Να καταναλώνεις 5 μερίδες φρούτων και λαχανικών ημερησίως.	Αυτή η συμβουλή είναι σημαντική για την υγεία

Διατροφικές συνήθειες και άλλες συμπεριφορές

- Οι θετικές στάσεις και προθέσεις για υγιεινή διατροφή επηρεάζουν και τις τελικές επιλογές για υιοθέτηση υγιεινών συνηθειών διατροφής των ατόμων. Όσο πιο θετικές στάσεις έχουν τα άτομα, τόσο πιο ισχυρή είναι και η αυτοπεποίθησή τους ότι μπορούν να ακολουθήσουν υγιεινές συνήθειες διατροφής.
- Η συμμετοχή σε φυσικές δραστηριότητες και προγράμματα άσκησης ενισχύει ακόμα περισσότερο την αυτοπεποίθηση και τις καλές διατροφικές συνήθειες των ατόμων. Όσο πιο σίγουρα είναι τα άτομα ότι μπορούν να ακολουθούν μία υγιεινή διατροφή, τόσο ισχυρότερες προθέσεις και στάσεις έχουν για την υγιεινή διατροφή και άσκηση ταυτόχρονα.
- Η προσέγγιση, λοιπόν, του ελέγχου του βάρους σχετίζεται με διαδικασίες αλλαγής και τροποποίησης στάσεων και προθέσεων των ατόμων, αλλά και σε θέματα υγιεινών συνηθειών γενικότερα. Στο παρακάτω σχήμα φαίνεται ότι οι κακές επιλογές οδηγούν σε παχυσαρκία και ασθένειες. Αντίθετα, η αλλαγή στάσεων και συμπεριφορών, η άσκηση και η υγιεινή διατροφή οδηγούν σε έλεγχο του βάρους και ποιότητα ζωής.

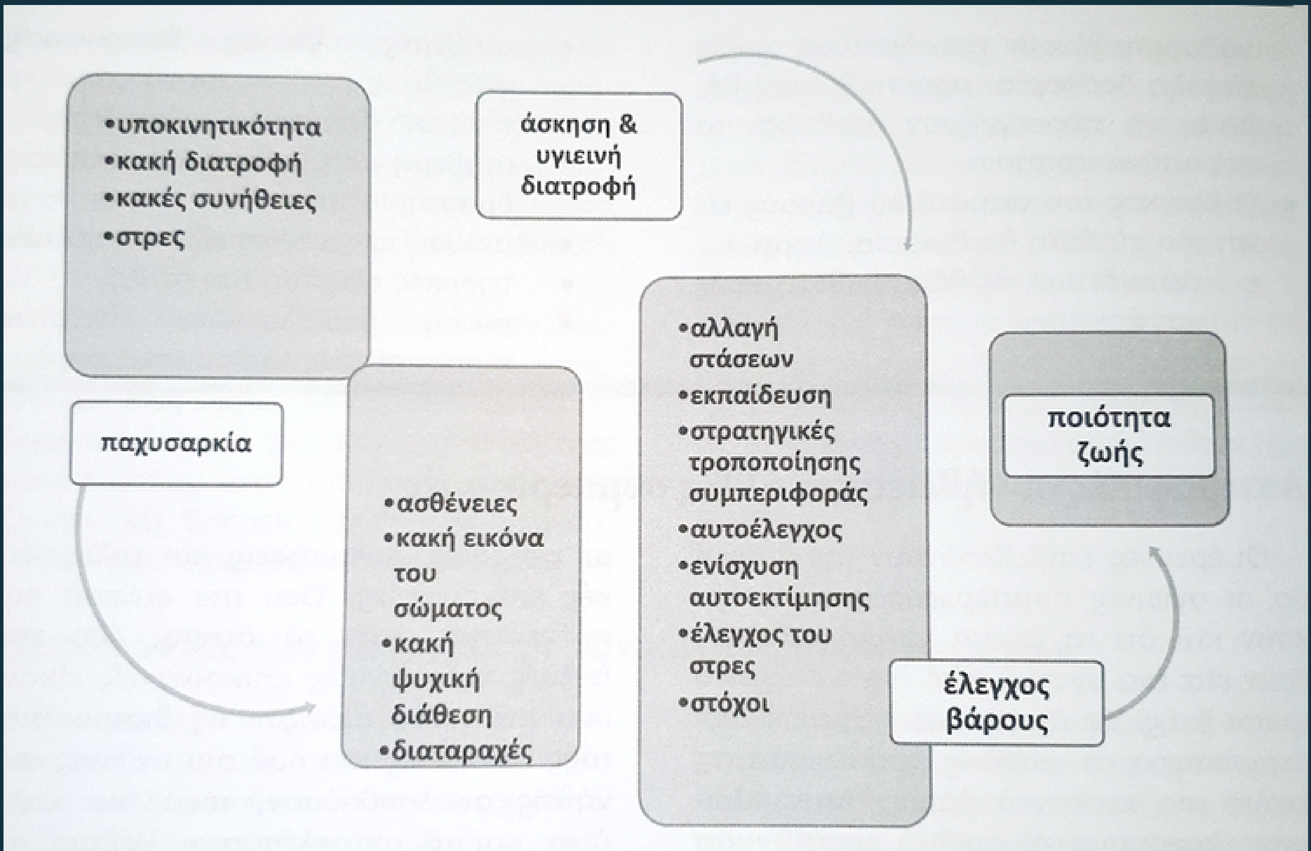

Σχήμα 13. *Ο έλεγχος της παχυσαρκίας οδηγεί σε ποιότητα ζωής*

Διατροφή και στρες

Σε περιόδους άγχους, η σχέση με την τροφή μπορεί να επηρεαστεί, οδηγώντας σε υπερβολική κατανάλωση. Τα άτομα που προσπαθούν να αδυνατίσουν δέχονται ισχυρές ψυχολογικές επιδράσεις. Αυξάνεται η ψυχολογική τους πίεση, το στρες, η κατάθλιψη και μειώνεται η αυτοεκτίμησή τους και η εικόνα του σώματός τους. Αντίθετα όταν το πρόγραμμα δίαιτας οδηγεί σε θετικά αποτελέσματα και μείωση του βάρους του σώματος, τότε οι παραπάνω δείκτες βελτιώνονται. Θεωρείται ότι η έλλειψη αυτοσυγκράτησης στην ποσότητα της τροφής οφείλεται περισσότερο στο στρες των ατόμων και όχι στην παχυσαρκία αυτή καθ' εαυτή. Άτομα με υψηλά επίπεδα νευρωτισμού και χαμηλά επίπεδα ευσυνειδησίας έχουν υψηλότερο δείκτη μάζας σώματος. Έτσι οι διαδικασίες επιδίωξης της ψυχικής ευεξίας και του ελέγχου του στρες, είναι αναγκαίες σε κάθε πρόγραμμα ενίσχυσης των υγιεινών συμπεριφορών και ελέγχου του βάρους.

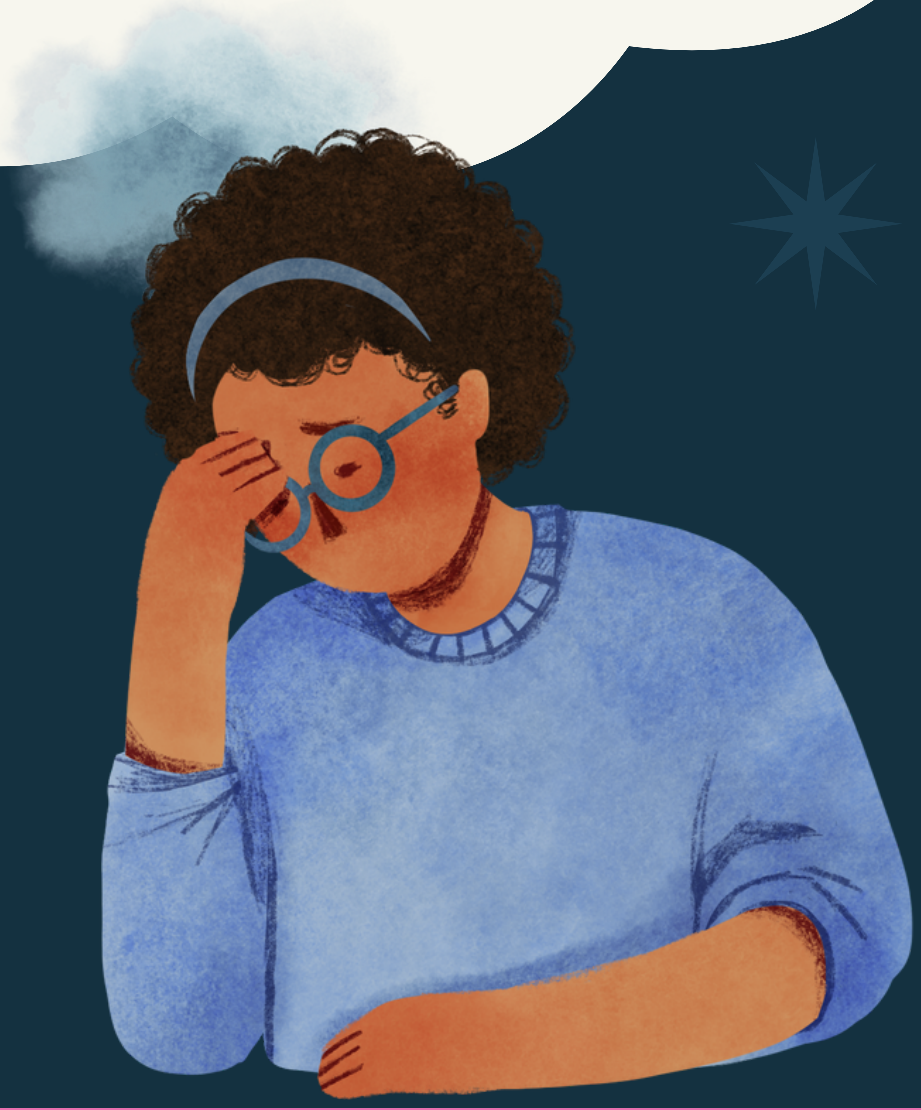

Τροποποίηση συμπεριφοράς

ΤΙ ΘΕΛΟΥΜΕ ΝΑ ΤΡΟΠΟΠΟΙΗΣΟΥΜΕ, ΤΙ ΘΕΛΟΥΜΕ ΝΑ ΠΕΤΥΧΟΥΜΕ	ΤΙ ΠΡΟΤΕΙΝΟΥΜΕ, ΠΟΥ ΕΣΤΙΑΖΟΥΜΕ, ΤΙ ΤΟΝΙΖΟΥΜΕ
ΠΕΡΙΒΑΛΛΟΝ	Δεν πλησιάζουμε σε μέρη – πειρασμούς που προσφέρουν ανθυγιεινές τροφές.
ΠΡΟΣΔΟΚΙΕΣ	Μέσα από την σωστή διατροφή και την άσκηση, προσδοκούμε μία καλύτερη ποιότητα ζωής.
ΠΡΟΤΥΠΑ	Σωστά πρότυπα είναι τα άτομα που τρέφονται υγιεινά και ασκούνται, αυτά θωρακίζουν καλύτερα την υγεία τους.
ΑΥΤΟ-ΑΠΟΤΕΛΕΣΜΑΤΙΚΟΤΗΤΑ	Σκεφτόμαστε νοερά πως θα είναι το σώμα και υγεία μας σε λίγο καιρό.
ΑΥΤΟ-ΠΑΡΑΚΟΛΟΥΘΗΣΗ	Καταγράφουμε σε μία λίστα τι φάγαμε κάθε μέρα και πόσο χρόνο αφιερώσαμε σε φυσικές δραστηριότητες.
ΔΕΣΜΕΥΣΗ	Συζήτηση, στο τέλος κάθε εβδομάδας, με ένα σημαντικό για εμάς πρόσωπο, σχετικά με όσα γράψαμε στην λίστα.
Η ΑΠΕΙΛΗ	Η παχυσαρκία είναι αιτία πολλών σοβαρών ασθενειών, είναι και η ίδια ασθένεια. Όσοι έχουν πρόβλημα παχυσαρκίας κινδυνεύουν.
ΤΑ ΟΦΕΛΗ	Η ρύθμιση του βάρους είναι ένας από τους δείκτες καλής υγείας. Το άτομο τότε λειτουργεί ευχάριστα και αποδοτικά και χαίρεται καλύτερα τη ζωή του.
ΤΑ ΕΜΠΟΔΙΑ	Δεν υπάρχουν εμπόδια στην ρύθμιση του βάρους, όταν το πρόβλημα δεν είναι παθολογικό. Είναι απλά θέμα απόφασης και συγκεκριμένων συνηθειών.
ΣΥΝΑΙΣΘΗΜΑΤΙΚΑ	Ευαισθητοποίηση των ατόμων με χρήση φωτογραφιών ή βίντεο, με παροχή πληροφοριών για ασθένειες που σχετίζονται με την κακή διατροφή.
ΓΝΩΣΤΙΚΑ	Γνώσεις σχετικά με την σωστή διατροφή και την αξία της άσκησης. Τονίζεται ο ρόλος του συνδυασμού άσκησης και σωστής διατροφής για καλή υγεία.
ΟΙ «ΣΗΜΑΝΤΙΚΟΙ ΑΛΛΟΙ»	Οι γονείς ενημερώνονται για την σωστή διατροφή των παιδιών τους. Η βελτίωση της εικόνας του σώματος και της εμφάνισης δημιουργεί αποδοχή και θαυμασμό των «σημαντικών άλλων».
Ο ΕΛΕΓΧΟΣ	Όλοι μπορούν να έχουν τον έλεγχο της διατροφής τους, αρκεί να πιστεύουν στην σημαντικότητα και στο ότι μπορούν να τα καταφέρουν να τρέφονται υγιεινά. Περιόρισε τα εμπόδια. Αύξησε τις ευκαιρίες. Δώσε δυνατότητες επιλογής.
ΣΥΜΠΕΡΙΦΟΡΕΣ	Υποδεικνύουμε άλλα πρότυπα. Καταγράφουμε καλές και κακές συνήθειες των άλλων και τις σχολιάζουμε.

Διατροφικές διαταραχές

Οι διαταραχές διατροφής χαρακτηρίζονται από την υπερβολική ενασχόληση με το σωματικό βάρος και την εικόνα του, συνοδευόμενες από ελλιπή, ακατάλληλη, ακανόνιστη ή υπερβολική πρόσληψη τροφής. Δύο είναι οι βασικές κατηγορίες διαταραχών διατροφής, η νευρική ανορεξία και η βουλιμία.

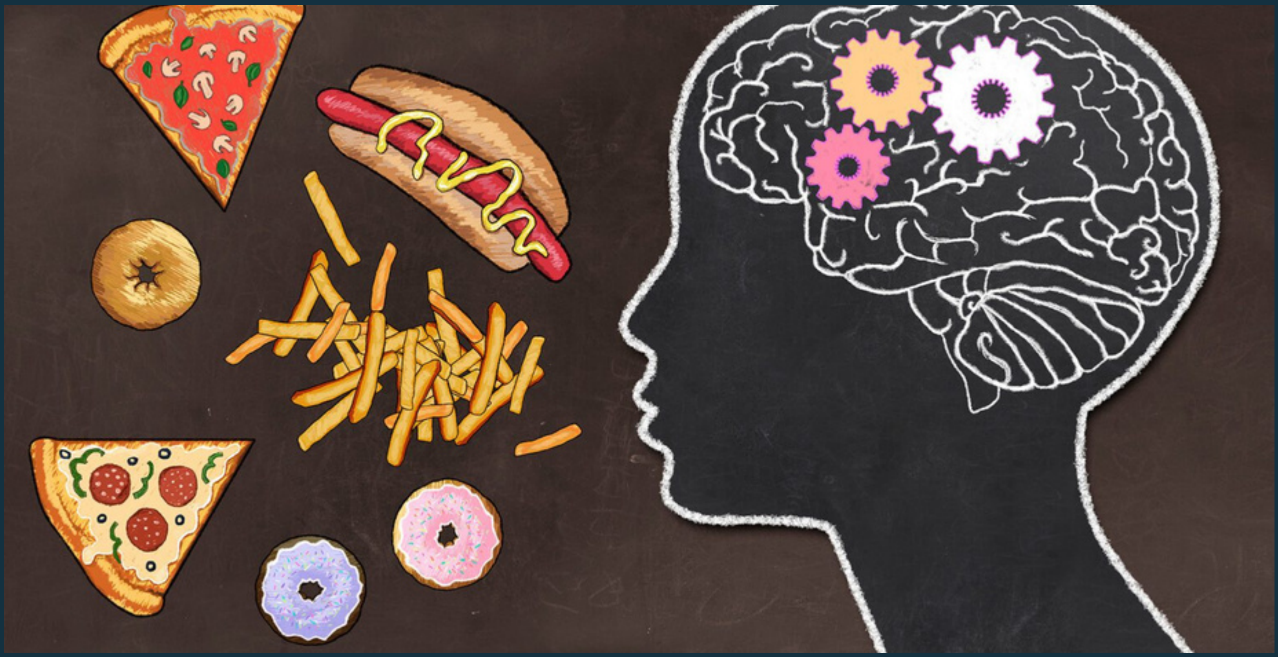

- Νευρική ανορεξία: Η νευρική ανορεξία έχει ψυχοφυσιολογική βάση και χαρακτηρίζεται από έντονο φόβο για παχυσαρκία, κακή εικόνα του σώματος, μεγάλη απώλεια βάρους, άρνηση για διατήρηση του σωστού βάρους του σώματος και αμηνόρροια για τις γυναίκες.
- Βουλιμία: Η βουλιμία χαρακτηρίζεται από ανεξέλεγκτη κατανάλωση ποσοτήτων τροφής, από προσπάθεια πρόκλησης εμετού, έντονη άσκηση ή χρήση άλλων μεθόδων για κατανάλωση των θερμίδων και έλεγχο του βάρους, φόβο για μη έλεγχο της κατάστασης και καταθλιπτική διάθεση.

Άσκηση και διατροφικές διαταραχές

- Οι διαταραχές διατροφής επηρεάζουν αρνητικά την ποιότητα ζωής των ατόμων.
- Κυρίως στις γυναίκες, η βουλιμία σχετίζεται με τα έντονα στρεσογόνα γεγονότα της ζωής.
- Όταν η δίαιτα για τον έλεγχο του βάρους είναι ανεπιτυχής, τότε δημιουργείται κατάθλιψη, ως αποτέλεσμα της αποτυχίας, της δυσφορίας, της απελπισίας και της αρνητικής αυτό-αξιολόγησης που κάνει το άτομο. Η προσπάθεια αυτή καταλήγει σε ανεξέλεγκτες ή ανθυγιεινές διατροφικές συνήθειες.
- Ένα άλλο θέμα που σχετίζεται με τις διατροφικές διαταραχές είναι η υπερβολική ή ψυχαναγκαστική άσκηση. Ενώ η άσκηση σε φυσιολογικά επίπεδα έχει πολλά οφέλη για τα άτομα, ο εθισμός, η υπερβολική ή ψυχαναγκαστική άσκηση οδηγεί σε πλήθος ψυχολογικών και φυσιολογικών προβλημάτων.

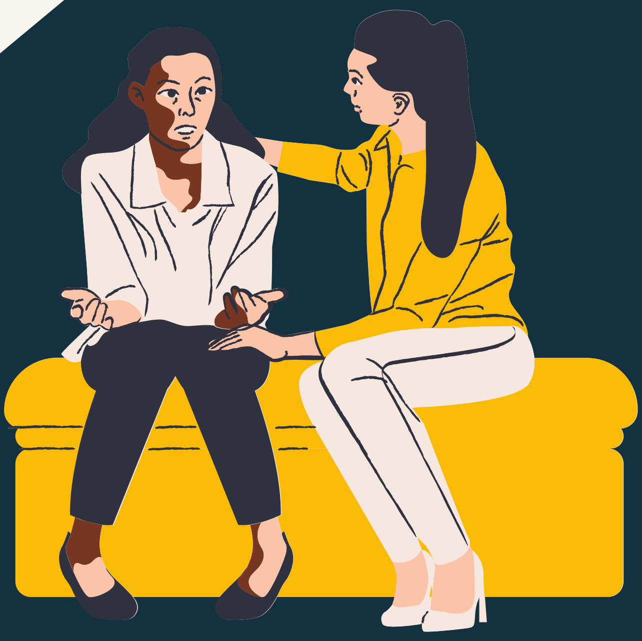

Οδηγίες για εφαρμογή

Άσκηση

- Τα όρια της διάρκειας της άσκησης για καλύτερα οφέλη στην υγεία και ιδιαίτερα στον έλεγχο του βάρους από τους σχετικούς επιστήμονες συνεχώς αυξάνονται. Προτείνουν, για παράδειγμα, συμμετοχή σε φυσικές δραστηριότητες μέτριας έντασης για περίπου 60 λεπτά την ημέρα, τις περισσότερες ημέρες της εβδομάδας.
- Ειδικότερα, για τον έλεγχο του βάρους προτείνεται από το American college of sports Medicine, άσκηση μέτριας έντασης από 150 έως 200 λεπτά την εβδομάδα ισοδυναμεί με 1200 έως 2000 kcal. Η άσκηση αυτής της ποσότητας έχει μέτρια αποτελέσματα στην μείωση του βάρους.
- Άσκηση μέτριας έντασης που διαρκεί από 225 έως 420 λεπτά την εβδομάδα οδηγεί σε σημαντική μείωση του βάρους. Ο συνδυασμός της άσκησης αυτής με την μείωση της κατανάλωσης θερμίδων (από 300 έως 600 kcal την ημέρα) έχει πιο καλά αποτελέσματα.
- Τα άτομα είναι καλό να επιλέγουν τον τύπο της άσκησης που επιθυμούν, να ρυθμίζουν μόνα τους την ένταση και να την αυξάνουν προοδευτικά, χωρίς μεγάλη δυσφορία, προσπαθώντας βέβαια, να προσεγγίζουν σταδιακά τα παραπάνω κριτήρια.

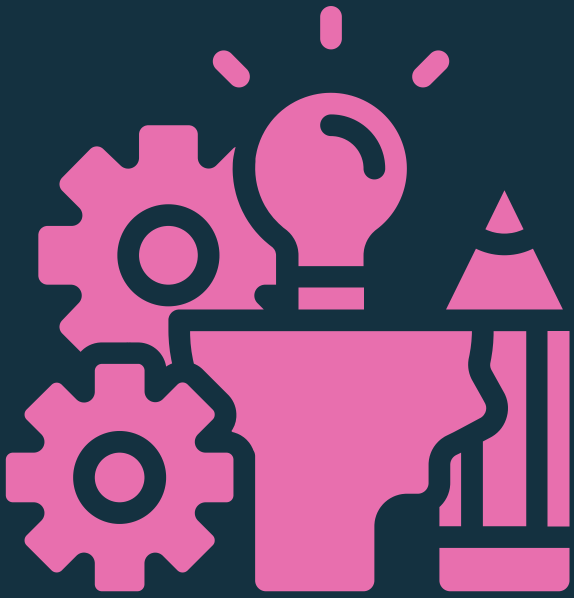

Οδηγίες για εφαρμογή

Έλεγχος του βάρους

- Τα σωστά προγράμματα ελέγχου του βάρους περιλαμβάνουν μέτρια μείωση της τροφής και προγράμματα αντοχής, με ταυτόχρονη τροποποίηση των συνηθειών διατροφής. Δηλαδή, μία μακροχρόνια δέσμευση σε κατάλληλες διατροφικές συνήθειες και άσκηση σε μόνιμη βάση.
- Τα γρήγορα προγράμματα δίαιτας είναι ακατάλληλα ή ιατρικά επικίνδυνα γιατί βασίζονται κυρίως σε μεγάλη μείωση της προσλαμβανόμενης τροφής και οδηγούν σε απώλεια μεγάλων ποσοτήτων ηλεκτρολυτών, νερού, μετάλλων και άλιπου ιστού, ενώ αντίθετα οδηγούν σε μικρές απώλειες του πραγματικού λίπους.

Διατροφικές συνήθειες

- Καλό είναι να ακολουθεί κανείς ισορροπημένη διατροφή με ποικιλία τροφών σε κάθε γεύμα, να μειώνει την πρόσληψη θερμίδων και να αυξάνει την κατανάλωση θερμίδων μέσω της άσκησης. Επίσης, να καταναλώνονται τροφές χαμηλές σε λιπαρά, με λιγότερη ζάχαρη και να αποφεύγεται το πολύ αλάτι. Σε πιο πρακτικό επίπεδο, καλό είναι να προτιμώνται χαμηλές σε θερμίδες τροφές, να καταναλώνεται μεγάλος όγκος διαφορετικών φυτικών ινών και μικρές μερίδες κρέατος. Τέλος, να αποφεύγεται το αλκοόλ και να καταναλώνονται μεγάλες ποσότητες υγρών κάθε μέρα, κατά προτίμηση νερού.

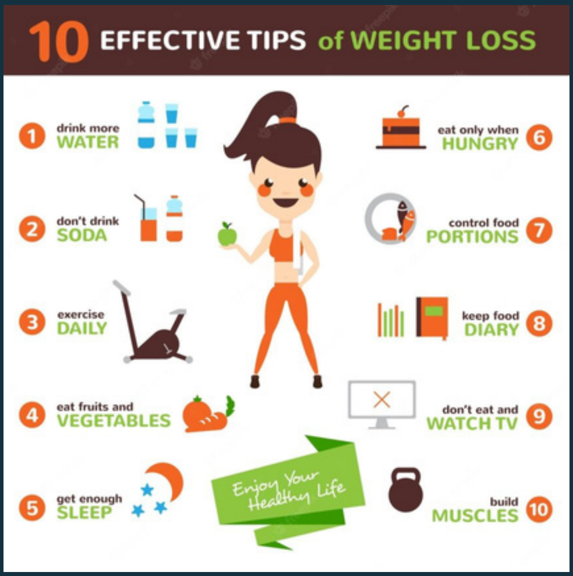

Οδηγίες για εφαρμογή

Προγραμματισμός

- Να εφαρμόζει τις αρχές της θεωρίας των στόχων ορίζοντας συγκεκριμένο αριθμό κιλών που θέλει να χάσει και συγκεκριμένο αριθμό θερμίδων ημερησίως. Να γράφει τις τροφές που καταναλώνει, να κάνει πρόγραμμα της ημερησίας κατανάλωσης τροφής, να κάνει ωρολόγιο πρόγραμμα των γευμάτων, να προετοιμάζει την λίστα των πραγμάτων που θα αγοράσει από το σουπερμάρκετ, να αγοράζει όταν δεν πεινάει, να αποφασίζει από πριν τι θα παραγγείλει όταν τρώει εκτός σπιτιού, να αποφεύγει μεγάλες περιόδους στέρησης πριν πάει να φάει εκτός σπιτιού, να σχεδιάζει μία μικρή καθυστέρηση προτού αρχίσει να τρώει, να αφήνει κάθε φορά λίγο φαγητό στο πιάτο του. Να μην υπάρχουν στο σπίτι πολλά φαγητά.
- Ο έλεγχος του σωματικού βάρους είναι μία σύνθετη διαδικασία. Απαιτεί εκπαίδευση σε θέματα διατροφής και άσκησης, σταθερές υγιεινές διατροφικές συνήθειες, κινητικά δραστήριο τρόπο ζωής, σταθερή συμμετοχή σε φυσικές δραστηριότητες, μείωση των καθιστικών συνηθειών, τεχνικές ελέγχου του στρες, γνώση ψυχολογικών τεχνικών τροποποίησης της συμπεριφοράς

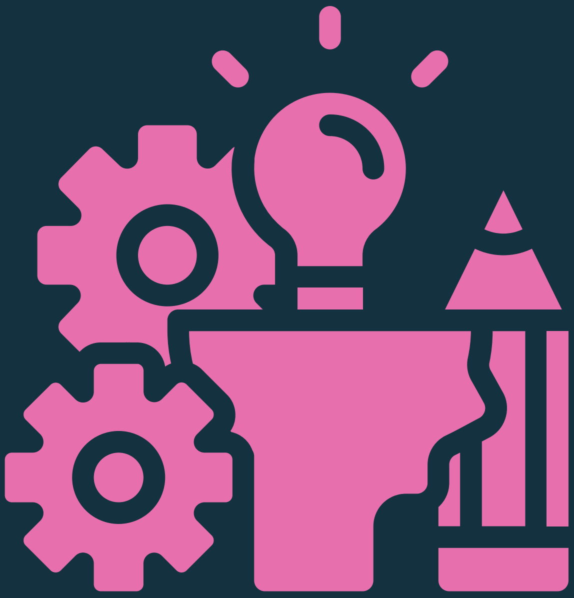

Οδηγίες για εφαρμογή

Καθημερινές συνήθειες

- Να αποφεύγει κανείς τους «πειρασμούς», να μην υπάρχουν πολλά φαγητά στο τραπέζι, να αδειάζει το τραπέζι αμέσως μόλις τρώει, σε γιορτές να μην πλησιάζει στα μέρη όπου έχει φαγητό. Να τρώει καθιστός, όχι όρθιος. Να τρώει αργά και χαλαρά. Να μην κάνει κάτι άλλο, όταν τρώει (όπως να μη βλέπει τηλεόραση). Να περιορίζει τα μέρη όπου τρώει (π.χ. κουζίνα ή εστιατόριο). Να ασχολείται με χόμπι και δραστηριότητες αναψυχής. Να αποφεύγει, όσο μπορεί, λεωφορεία, αυτοκίνητο, ασανσέρ. Να προτιμά το περπάτημα.

Αυτό-παρακολούθηση

- Να χρησιμοποιούνται βηματόμετρα ή άλλα συναφή φορητά όργανα, GPS η επιταχυνσιόμετρα, για την καταγραφή των βημάτων, της απόστασης που διανύει κάποιος, της συχνότητας των σφυγμών, της συνολικής φυσικής δραστηριότητας σε μία μέρα, ημερολόγια καταγραφής της ποσότητας και της ποιότητας της άσκησης, ημερολόγια καταγραφής θερμίδων κλπ.

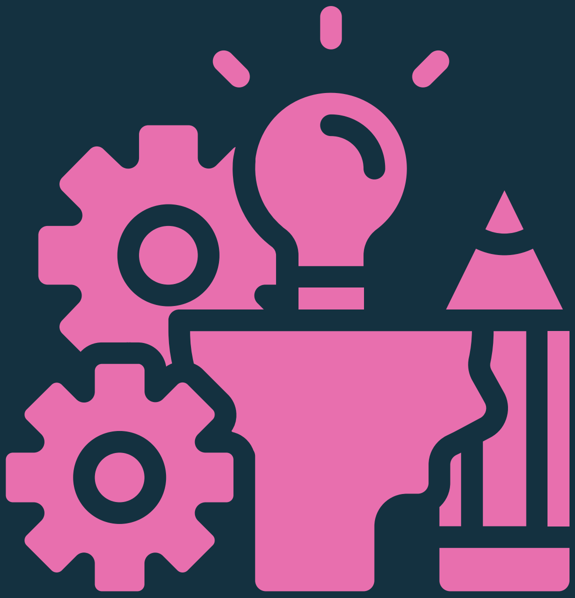

Επίλογος

Η παχυσαρκία επηρεάζει αρνητικά την ποιότητα ζωής. Ο έλεγχος του βάρους και η αποφυγή της παχυσαρκίας και των διατροφικών διαταραχών, απαιτούν έναν ισορροπημένο, υγιεινό τρόπο ζωής. Η ισορροπία αυτή επιτυγχάνεται με τη γνώση των διατροφικών συνηθειών, με υγιεινή διατροφή, με συστηματική άσκηση και γενικά με συμμετοχή σε φυσικές δραστηριότητες. Ο έλεγχος του βάρους διαμέσου της άσκησης είναι ο ασφαλέστερος τρόπος, καθώς έτσι, τα αποτελέσματα είναι εμφανέστερα, τα οφέλη στην υγεία είναι πολλαπλάσια και η θετική ψυχική διάθεση που αναπτύσσεται ενισχύει την προσπάθεια των ατόμων για μακροχρόνια δέσμευση και μόνιμη αλλαγή του τρόπου ζωής.

Πηγές

Θεοδωράκης, Γ. (2017). Άσκηση, Ψυχική Υγεία και Ποιότητα Ζωής. (Δεύτερη αναθεωρημένη έκδοση, σελ. 149-170). Εκδόσεις Αφοι Κυριακίδη.

Γεροδήμος, Β. (2013) Η άσκηση ως μέσο πρόληψης και αποκατάστασης χρόνιων παθήσεων. Προοπτική Ε.Π.Ε. Πασχάλης, Β., Νικολαΐδης, Μ., Δίπλα, Κ., Τζιαμο΄θερτας, Α., Καρατεράντου Κ., Μελισσοπούλου, Α. & Γεροδήμος, Β. Άσκηση και παχυσαρκία, σελ 262- 288.

Beeken, R.J., Croker, H., Morris, S. et al. Study protocol for the 10 Top tips (10TT) trial: Randomised controlled trial of habit-based advice for weight control in general practice. BMC Public Health 12, 667 (2012). https://doi.org/10.1186/1471-2458-12-667
