# Supplementary material for: Effectiveness and acceptance of virtual reality vs. traditional exercise in obese adults: a pilot randomized trial
Source: Front Sports Act Living. 2025 Mar 19;7:1520068. doi: 10.3389/fspor.2025.1520068 (PMC11962008; doi:10.3389/fspor.2025.1520068)
Supplement: Supplementary file 5 [file Table1.docx]

Supplementary Material

Supplementary Material 1. Brochure

**Supplementary Material 2.** SDT behavior change techniques according to Teixeira et al., 2019, used and applied in the intervention

| ***Behavior change technique*** | ***Applied in the intervention*** |
| --- | --- |
| **Autonomy support** | |
| Elicit perspectives on condition or behavior | Using the TPB questionnaire and the first session of counseling to investigate the participants’ perspectives toward participation in exercise. |
| Prompt identification of sources of pressure for behavior change | During the first counseling session, a discussion was held to explore the participants' motivations for engaging in exercise as well as any sources of pressure they may be experiencing. |
| Use non- controlling, informational language | During the counseling sessions, non-controlling language was employed. For example, words like "might" or "could" were used to suggest potential strategies for overcoming obstacles and engaging in exercise and diet programs. |
| Provide choice | All participants could freely choose their preferred form of exercise, including the frequency, duration, and intensity level. They could also tailor the exercise program to fit seamlessly into their daily routines. |
| Encourage the person to experiment and self-initiate the behavior | Experiment and self-initiate were facilitated through participation in the VR-exercise program, but also in traditional exercise as they could manage their exercise program |
| **Relatedness support** | |
| Acknowledge and  respect perspectives  and feelings | Statements of empathy, support and motivation were provided through Viber chat and counseling sessions to create a warm and accepting environment |
| Encourage asking of questions | They were free to ask questions anytime through Viber chat and during counseling sessions |
| Show unconditional regard | Positive support was provided during counseling sessions, regardless of participants' outcomes in the exercise or diet program. |
| Demonstrate/show  interest in the person | Statements of interest about the participants’ thoughts and perceptions were provided in counseling sessions |
| Use empathic listening | Participants had time to express themselves without pressure in counseling sessions |
| **Competence support** | |
| Address obstacles for change | Discussion of likely barriers in behavior change in the counseling session. The booklet given was designed to provide relevant information and help participants to deal with most common barriers. |
| Clarify expectations | Discussion of their expectations and goal-setting during counseling sessions |
| Assist in setting optimal challenge | Education about SMART goal-setting was provided through first counseling session |
| Offer constructive, clear, and relevant feedback | Counseling sessions included discussions on whether goals were achieved or not, with adjustments made to the goals as needed. |
| Help develop a clear and concrete plan of action | Discussion about the changes they need to make in order to reach the goals they have yet to achieve and goal adjustments |
| Promote self-monitoring | Self-monitoring forms were a used as a tool to help self-monitoring |
| Explore ways of dealing with pressure | The booklet provided to participants was designed to support them in overcoming barriers and managing pressures related to maintaining a healthy lifestyle. It offered practical advice on how to navigate challenges such as adhering to a balanced diet and staying consistent with exercise routines. Additionally, it included strategies for coping with stress, anxiety, and other pressures that may interfere with their health goals. |

**Supplementary Material 3.** Booklet

**Supplementary Material 4.** Daily Self-Monitoring Form

**Supplementary Material 5.** Goal-Setting Form

**Supplementary Material 6.** PowerBeatsVR Trailer <https://www.youtube.com/watch?v=ATXOTp7NaQM>

**Supplementary Material 7.** Participant’s screen video while exercising with PowerBeatsVR (video)

**Supplementary Material 8.** Thematic analysis from the semi-structured interview for the intervention group

| Question | Answer | Frequency |
| --- | --- | --- |
| 1.Can you share your experience from your participation in this study? | Innovative, highly entertaining, with a positive influence on my psychology and motivation for physical activity | 30% |
|  | Extremely fun, interesting and innovative, the support and guidance I received was important | 25% |
|  | I am grateful to try this type of exercise and achieve my goals | 25% |
|  | Innovative, pleasant, comfortable, easy, accessible and attractive form of exercise | 20% |
| 2. In your opinion, how your participation in the program helped you to change your behavior regarding exercise and nutrition? | Enhancing my consistency in the program, understanding the importance of determination and concentration to my goal | 30% |
|  | Changed my perspective on exercise and nutrition | 30% |
|  | It helped me to improve my psychology and organize myself | 15% |
|  | By understanding the value of exercise and creating ways to deal with obstacles | 15% |
|  | With the constant support and the very enjoyable type of exercise I felt that I could incorporate exercise into my daily life | 15% |
| 3. How would you describe your belief that you can do it (self-efficacy) about exercise before and after the intervention? | The intervention helped me to recognize my potential and my self-efficacy improved | 60% |
|  | My self-efficacy levels were high, but it was enhanced until the end of the intervention | 30% |
|  | Fear of failure has been decreased | 10% |
| 4. How did you manage the challenges, concerns or obstacles that arose during the program? | With great support and communication with the researcher | 40% |
|  | With composure and persistence in my goals | 30% |
|  | I didn't face any problems | 20% |
|  | The digital booklet and online communication helped a lot | 20% |
| 5. What do you think would help you do better? | If I had more free time | 30% |
|  | If I could practice more in the VR environment and improve my performance | 25% |
|  | I believe I have fulfilled the desired objectives to the fullest extent | 20% |
|  | If I had more energy | 10% |
|  | If there were other VR users to practice with | 15% |
| 6. What changes did you notice in your mood during this effort? | I was in a better mood, more optimistic and had a sense of wellness | 50% |
|  | I felt joy and motivated regarding exercise | 35% |
|  | My mood was improved even on “bad” days | 20% |
|  | I felt great self-confidence | 20% |
|  | I felt a sense of wellness and relaxation, reduced anxiety and stress | 15% |
|  | I felt full of energy and vitality | 10% |
| 7. How did the program help you to take care of your body? | My body image was positively affected | 45% |
|  | Because I participated in the exercise program, I have decided to follow a diet program to take better care of my body | 45% |
|  | I take care of my body as I always did | 10% |
| 8. What exercise and diet tips did you find helpful in the digital booklet you received? | All of them were useful and interesting | 50% |
|  | Tips for managing stress and anxiety through exercise | 20% |
|  | The importance of dedication in the program and patience | 20% |
|  | Tips for exercise and active lifestyle through daily choices | 15% |
|  | Tips about overeating and emotional eating | 10% |
| 9. How did the process of right goal-setting help you? | With the SMART method, I learned to set goals in a correct way | 80% |
|  | It helped me to properly assess my needs | 40% |
|  | Given goal-setting forms and researcher’s guidance helped me | 35% |
| 10. How did the process help you adjust your goals each week? | I evaluated my progress and set new, more challenging goals | 90% |
|  | With the goal-setting forms given | 25% |
|  | The advice from the researcher and constant motivation helped me to adjust my goals | 25% |
| 11. How has self-monitoring your progress helped you achieve your goals? | I could better evaluate my progress and my self-confidence was increased | 40% |
|  | I avoided "breaking out" of the program because I didn't want to write it | 25% |
|  | It helped me focus on my goals | 25% |
|  | I could monitor my mistakes and try to correct them | 15% |
|  | I felt confident and secure because I felt in control | 10% |
| 12. On a scale of 1 to 10, how much do you think you intend to continue exercising with the same or more frequency? | 10 | 35% |
|  | 9 | 25% |
|  | 8 | 20% |
|  | 6 | 10% |
|  | 7 | 5% |
|  | 5 | 5% |
| 13. What would make you score higher than the one you mentioned above? | If I had more free time/ less obligations | 35% |
|  | Nothing, I scored 10 | 35% |
|  | More motivation and rewards | 15% |
|  | If I could have access to the VR system whenever I wanted | 10% |
|  | If I liked working out and had company while exercising | 5% |
| 14. On a scale of 1 to 10, how much do you think you intend to continue using the behavior change techniques you implemented in this program? | 10 | 35% |
|  | 8 | 25% |
|  | 9 | 20% |
|  | 7 | 10% |
|  | 5 | 5% |
|  | 6 | 5% |
| 15. What would make you score higher than the one you mentioned above? | Nothing, I scored 10 | 35% |
|  | If there was someone to motivate and guide me | 30% |
|  | My participation in a community with people with similar goals with me | 20% |
|  | If I had access to an application which could give me feedback for my progress and encourage me to use such techniques | 15% |
| 16. How do you feel about the support and guidance you received to manage challenges relevant to adopting healthy habits? | Very good, it was very useful | 50% |
|  | It helped me to stay focused on my goals | 20% |
|  | I felt that I wasn’t alone and I had guidance | 20% |
|  | It was maybe the most basic thing | 10% |
| 17. How did the online communication help you overcome challenges or obstacles that may have arisen? | I felt that there was always someone who can guide, motivate and advise me | 50% |
|  | It guided me to remember my incentive | 25% |
|  | It helped me to adjust my goals | 15% |
|  | I didn’t need it | 10% |
| 18. On a scale of 1 to 10, how much did using the virtual reality app contribute to being more physically active this month? | 10 | 65% |
|  | 9 | 20% |
|  | 8 | 10% |
|  | 7 | 5% |
| 19. How do you think being able to choose the duration and intensity of your exercise program helped you to be more physically active this month? | I could adapt it to my abilities and reduced the stress of failure | 40% |
|  | I liked that I could control my exercise program | 30% |
|  | It helped me to choose the exercise factors according to the situation I was in that particular day | 25% |
|  | It didn't help me; I would like more challenging goals from external source | 5% |
| 20. How do you intend to continue with exercise in the future? | I would like to continue practicing with the VR | 60% |
|  | At the same frequency and intensity | 35% |
|  | I will increase exercise frequency and intensity | 25% |
|  | I will try to exercise as much as I can | 25% |
|  | I will join gym | 15% |
| 21. How did you benefit from the counseling sessions conducted during the study? | I have learned to set goals | 45% |
|  | I have learned important information about exercise and nutrition | 35% |
|  | I realized how important psychological support is for achieving goals | 20% |
|  | I found great motivation to exercise and follow a diet plan | 15% |

**Supplementary Material 9.** Thematic analysis from the semi-structured interview for the control group

| Question | Answer | Frequency |
| --- | --- | --- |
| 1.Can you share your experience from your participation in this study? | Positive and interesting experience | 75% |
|  | Counseling helped me to set goals and achieve them regarding exercise and diet | 40% |
|  | Pleasant and efficient | 25% |
| 2. In your opinion, how your participation in the program helped you to change your behavior regarding exercise and nutrition? | It helped me to change habits step by step through goal-setting | 25% |
|  | It helped me discipline myself through goal-setting | 25% |
|  | Helped me improve my diet and increase my exercise participation | 20% |
|  | It helped me because I had someone to guide me in the right direction | 10% |
|  | It helped me overcome difficulties | 10% |
|  | I learned a lot about exercise and nutrition and tried to improve my daily life and health | 10% |
| 3. How would you describe your belief that you can do it (self-efficacy) about exercise before and after the intervention? | The intervention helped me to recognize my potential and my self-efficacy improved | 80% |
|  | I didn't notice any difference | 20% |
| 4. How did you manage the challenges, concerns or obstacles that arose during the program? | Communication with researcher helped me a lot | 35% |
|  | With optimism and persistence in my incentive | 30% |
|  | With composure, self-restraint and discipline | 20% |
|  | I didn't face any problems | 15% |
| 5. What do you think would help you do better? | If I had more free time | 40% |
|  | Closer monitoring by exercise and nutrition experts | 25% |
|  | To be able to access public sports facilities (flexible hours) | 20% |
|  | If my health was better (weight, fitness, injuries) | 10% |
|  | If I had more appetite and will | 5% |
| 6. What changes did you notice in your mood during this effort? | I was in a better mood | 40% |
|  | I felt optimistic and a sense of wellness | 40% |
|  | I felt full of energy and vitality | 15% |
|  | I felt calmer | 10% |
|  | I was in a better mood, but I also felt some pressure not to deviate from the program | 10% |
| 7. How did the program help you to take care of your body? | My body image was positively affected | 50% |
|  | I take care of my body as I always did | 35% |
|  | I realized that physical self-care is very important | 15% |
| 8. What exercise and diet tips did you find helpful in the digital booklet you received? | All of them were useful and interesting | 50% |
|  | Tips for exercise and exercise programs | 20% |
|  | Helped me manage overeating and emotional eating | 10% |
|  | Tips about diet and usefulness of drinking water | 10% |
|  | Ideas about behavior change | 5% |
| 9. How did the process of right goal-setting help you? | With the SMART method, I learned to set goals in a correct way | 65% |
|  | It helped me to properly assess my needs | 20% |
|  | It helped me to prove to myself that it is not difficult to set goals and achieve them | 15% |
| 10. How did the process help you adjust your goals each week? | I evaluated my progress and set new, more challenging goals | 55% |
|  | With the goal-setting forms given | 35% |
|  | The advice from the researcher and constant motivation helped me to adjust my goals | 10% |
| 11. How has self-monitoring your progress helped you achieve your goals? | I could better evaluate my progress | 40% |
|  | It helped me focus on my goals | 25% |
|  | I could monitor my mistakes | 25% |
|  | I realized the changes I made in my daily life | 10% |
| 12. On a scale of 1 to 10, how much do you think you intend to continue exercising with the same or more frequency? | 10 | 30% |
|  | 8 | 20% |
|  | 7 | 20% |
|  | 5 | 15% |
|  | 9 | 10% |
|  | 4 | 5% |
| 13. What would make you score higher than the one you mentioned above? | If I had more free time | 45% |
|  | Nothing, I scored 10 | 30% |
|  | A bigger challenge | 10% |
|  | If I had company while exercising | 10% |
|  | If I liked working out | 5% |
| 14. On a scale of 1 to 10, how much do you think you intend to continue using the behavior change techniques you implemented in this program? | 10 | 30% |
|  | 8 | 25% |
|  | 7 | 15% |
|  | 5 | 10% |
|  | 9 | 5% |
|  | 6 | 5% |
|  | 3 | 5% |
|  | 4 | 5% |
| 15. What would make you score higher than the one you mentioned above? | If I had fewer obligations in my daily life | 35% |
|  | Nothing, I scored 10 | 30% |
|  | If there was someone to motivate me | 25% |
|  | I feel that I still need guidance | 10% |
| 16. How do you feel about the support and guidance you received to manage challenges relevant to adopting healthy habits? | Very good, it was very useful | 95% |
|  | It helped me to stay focused on my goals | 5% |
| 17. How did the online communication help you overcome challenges or obstacles that may have arisen? | It guided me to remember my incentive | 35% |
|  | I felt that there was always someone who can guide and advise me | 35% |
|  | It helped me because it was immediate and not time consuming | 15% |
|  | I didn’t need it | 15% |
